# Predicting depth of anaesthesia from single-channel EEG using a deep TCN-BiLSTM-attention model with EWMA

**DOI:** 10.1038/s41598-026-54608-8

**Published:** 2026-06-04

**Authors:** Sukriti, Chirag Kriplani, Suman Kumar, Abhishek Singh

**Affiliations:** School of Electronics Engineering, Vellore Institute of Technology, Chennai, India

**Keywords:** Depth of Anaesthesia, EEG, Bispectral Index Regression, Temporal Convolutional Network, Bidirectional LSTM, Exponentially Weighted Moving Average, Computational biology and bioinformatics, Engineering, Health care, Mathematics and computing, Medical research, Neuroscience

## Abstract

Accurately determining the depth of anaesthesia (DoA) is crucial for ensuring patient safety and providing individualised anaesthetic management. Widely used monitors, such as the Bispectral Index (BIS), rely on proprietary multichannel EEG algorithms, which limit transparency and accessibility. This study presents a single-lead EEG framework for continuous BIS estimation that is window-level causal, end-to-end, and suitable for near–real-time deployment. The approach integrates a Temporal Convolutional Network (TCN) with power-of-two dilations, a compact bidirectional LSTM for temporal refinement, and additive attention pooling, followed by an exponentially weighted moving average (EWMA) to stabilize predictions over time. This design captures multi-scale temporal dependencies directly from raw EEG while preserving interpretability and low latency. Using a random segment-level split on a public perioperative EEG–BIS dataset, the proposed model achieved a mean absolute error (MAE) of 4.499, root mean square error (RMSE) of 7.499, Pearson’s correlation coefficient (r) of 0.903, and Lin’s concordance correlation coefficient (CCC) of 0.897 relative to reference BIS values. Under a stricter subject-independent 5-fold GroupKFold evaluation, performance decreased as expected. Still, it remained stable across folds, with the best configuration achieving an MAE of 6.03 ± 0.38 and a CCC of 0.819 ± 0.050 after EWMA smoothing. With approximately 1.07 million parameters and a throughput of about 430 segments per second, the proposed framework offers an efficient and transparent solution for single-sensor DoA monitoring. Overall, this work advances data-driven BIS estimation beyond feature-based methods while explicitly addressing both within-dataset performance and generalization to unseen subjects.

## Introduction

Administering anaesthesia is a cornerstone of modern medicine, allowing patients to undergo surgical procedures safely and with minimal pain or discomfort. The Depth of Anaesthesia (DoA) reflects how strongly a general anaesthetic suppresses central nervous system (CNS) activity. Careful titration is essential, as errors in dosing carry distinct risks^1^. Administering too much anaesthesia can lead to cardiovascular or respiratory depression, delayed emergence, and prolonged recovery, and has been linked to higher risks of neurological injury and postoperative infection^2^. In contrast, administering too little increases the likelihood of patient movement, hemodynamic instability, and intraoperative awareness, which can have lasting psychological consequences^3^. In routine practice, anaesthesiologists infer DoA from clinical signs, changes in heart rate, blood pressure, respiration, sweating, tearing, pupillary response, and temperature, integrated with clinical judgment^4^. These physiological indicators vary substantially with patient factors (age, body weight, sex, comorbidities) and surgical stimuli, rendering consistent titration challenging^5^.

Because general anaesthetics primarily act on the brain, electroencephalography (EEG) provides a direct, non-invasive window into cortical dynamics under anaesthesia^6^. Relative to the low-amplitude, high-frequency patterns of wakefulness, anaesthetic exposure produces characteristic changes in EEG frequency content and waveform morphology that depend on both the pharmacologic agent and the stage of anaesthesia^7,8^. These properties motivated EEG-derived clinical monitors such as the Bispectral Index (BIS), Auditory Evoked Potentials, Narcotrend, Entropy Index, and Cerebral State Index^9^. Among these monitors, BIS is the most widely used, condensing processed EEG into a 0–100 index of cortical anaesthetic effect. The mapping commonly used is: BIS > 80 = awake, 60–80 = light sedation, 40–60 = general anaesthesia, and < 40 = deep anaesthesia^10^. Although BIS-guided care has been associated with moderate reductions in anaesthetic use, postoperative nausea/vomiting, and recovery time, several practical barriers remain^11^. In many settings, hardware and disposables are costly, and the algorithm is proprietary and undisclosed, creating “black-box” readings that are difficult to interpret when the number conflicts with the clinical picture^12^. In addition, EEG-based indices are susceptible to various common operating-room influences: physiological conditions such as hypothermia, hypotension, hypoxia, and metabolic disturbances can diminish or slow EEG activity, falsely indicating a deeper level of anaesthesia. Conversely, technical interferences like electromyographic signals from jaw tension or shivering, poor electrode contact, and electrocautery can simulate cortical activation, resulting in falsely elevated EEG index values^13,14^. Because these problems can cause clinicians to either chase false alarms or delay responding to genuine changes, trust in such systems often suffers, making them less likely to be used consistently. This is why there’s strong motivation to develop open, data-driven methods that utilise EEG features more effectively, reliably account for individual differences, and deliver stable performance in near-real-time clinical monitoring^15^. These newer techniques focus on working directly with raw or minimally processed EEG data using advanced machine learning or deep learning models. The goal is to create monitoring tools that are more transparent, adaptive, and less vulnerable to artefacts, with the potential to support broader clinical applicability, rather than relying on opaque proprietary indices.

Existing methods for EEG-based anaesthesia monitoring span both classification and regression frameworks. On the classification side, both feature-engineered machine learning (ML) models and end-to-end deep learning (DL) models have been used: Nsugbe & Connelly^16^ compared five pipelines: Noise-Assisted Empirical Mode Decomposition (EMD), raw-signal features, Linear Series Decomposition Learner, Deep Wavelet Scattering, and DL features; the simplest one, including raw signal, enhanced features, and Linear Discriminant (LDA) classifier, performed best. Liu et al.^17^ utilized Convolution Neural Networks (CNNs) trained on Short-Time Fourier Transform (STFT) spectrograms to model consciousness levels. In^18^, the authors introduced Ensemble EMD-derived spectral images that classified anaesthetic depth well with CNNs. Huang et al.^19^ proposed single-channel transformer models that fuse spectral and entropy features to classify anaesthesia states. These approaches reliably separate discrete stages or BIS ranges but do not provide the continuous estimates needed for fine-titration and closed-loop control, motivating a shift toward continuous BIS regression on larger, more diverse cohorts.

In comparison, regression-based approaches estimate continuous BIS values directly from EEG signals or derived features, providing a more granular representation of anaesthetic depth. Among traditional regression methods, Liu et al.^20^ combined sample entropy and multivariate empirical mode decomposition (EMD) with a random forest (RF) regressor in a cohort of 24 subjects, demonstrating the feasibility of continuous DoA tracking. Their approach achieved superior accuracy compared to recurrence analysis, permutation entropy, neural networks, and SVM-based models, with a Pearson’s correlation coefficient of $\:r=0.90\pm\:0.05$ and a mean absolute error (MAE) of $\:5.22\pm\:2.12$. Alsafy and Diykh^21^ proposed a single-channel EEG framework that used sliding-window segmentation, hierarchical dispersion entropy features, and community graph-based feature selection for BIS estimation. Their model achieved a correlation coefficient of 0.96. Schmierer et al.^22^ proposed SSEDoA, a DoA index designed using empirical wavelet transform (EWT), spectral entropy (SE) features, and a second-order difference plot, classified with a linear SVM. Using single-channel EEG from nine patients, SSEDoA showed strong agreement with BIS (Pearson *r* = 0.834) and high concordance for stage transitions (Cohen’s κ = 0.809), indicating effective tracking across the various phases of anaesthesia. Ra et al.^23^ computed SE features on pre-denoised EEG divided into ten frequency bands and found that SE from the β–γ (21.5–38.5 Hz) and β bands had the strongest association with BIS, with R^2^=0.8458 and R^2^=0.7312, respectively. Shin et al.^24^ computed phase lag entropy (PLE) from four-channel EEG in 35 adults under propofol TCI and compared it with BIS: PLE correlated strongly with BIS during induction (R^2^=0.84), matched BIS at loss of consciousness (PLE 62.3 vs. BIS 61.8; difference not statistically significant), and was lower at recovery (PLE 64.4 vs. BIS 75.7; *p*<0.05). Using single-channel EEG from 56 patients, Chen et al.^25^ introduced EEG variability (EEGV), defined as the fluctuation in the interval between successive local maxima. They derived eight parameters in the time and frequency domains. Compared with conventional features such as sample entropy, permutation entropy, median frequency, and spectral edge frequency, EEGV metrics showed higher correlations with BIS and significantly better discrimination between awake and anaesthesia states (*p* < 0.05), indicating improved tracking of DOA with a single lead. Shahbakhti et al.^26^ proposed parameter-free features for single frontal EEG DoA monitoring. After denoising and subband decomposition, features like entropy, power–frequency, fractal, and variation features are extracted, and the most informative are fed to an RF regressor to estimate the DoA index. The method achieved correlation coefficients of 0.80 and 0.79, with MAEs of 7.1 and 9.0, on the two datasets, respectively. Shahbakhti et al.^27^ fused fuzzy entropy with Gaussian and exponential memberships for a wearable single frontal EEG system, achieving a mean * r* = 0.85 to BIS and MAE = 5.4, outperforming sample and approximate entropy.

DL advances enable end-to-end feature extraction and regression with improved performance and generalizability. Afshar et al.^28^ designed a combinatorial DL structure combining inception CNN modules, bidirectional LSTM, and an attention mechanism, yielding RMSE 5.59 ± 1.04 and MAE 4.3 ± 0.87 on a large clinical dataset, outperforming feature-based and ResNet models. This architecture captures both spatial and temporal EEG patterns for accurate continuous BIS regression and multi-class classification (accuracy 88.7%). Another study by Afshar and Boostani^29^ proposed a two-stage DL scheme with ResNet CNN and BiLSTM, achieving MAE ~ 4.6 to 5 and balanced regression-classification performance, validating on 176 subjects’ forehead EEG. Zhang et al.^30^ introduced FEHANet, a frequency-enhanced hybrid attention network that mines multi-band Fourier spectral features with attention modules, achieving a mean squared error of approximately 6.18 and a classification accuracy of nearly 89.6% on a large public VitalDB dataset, thereby demonstrating improved frequency-band feature learning. Hwang and colleagues^31^ developed an interpretable deep learning model to predict BIS values by breaking down EEG signals into amplitude and phase components using the fast Fourier Transform (FFT). They combined this with an attention mechanism that highlights important features, achieving an RMSE of 6.6 and an area under the receiver operating characteristic curve (AUROC) of 0.94.

More broadly, recent EEG deep-learning research has expanded beyond task-specific CNN/RNN pipelines toward models that explicitly capture temporal-spectral structure, long-range dependencies, and transferable representations. For motor-imagery decoding, BCINetV1^32^ integrates temporal and spectral attention, illustrating the value of jointly modeling these two dimensions in EEG analysis. At a larger scale, LaBraM^33^ and EEGPT^34^ investigate pretrained EEG representation learning across datasets and downstream tasks, reflecting the growing interest in transferable EEG feature learning. Transformer-based EEG models have also been used to model long-range spatiotemporal dependencies, as illustrated by B2-ViT Net^35^ for seizure prediction. Hybrid architectures that combine convolutional, recurrent, and attention mechanisms have similarly been applied to physiological-state assessment, including EEG-based pilot-fatigue detection^36^. Recurrent sequence modeling also remains relevant in EEG-based state monitoring outside anaesthesia, as shown by recent LSTM-based driver-fatigue recognition work^37^. In contrast, earlier EEG studies in related application areas often relied on handcrafted band-power and statistical features combined with conventional machine-learning classifiers^38^. Taken together, these studies reflect the broader shift from feature-engineering-based EEG analysis toward learned temporal and spatiotemporal representations.

At the same time, BIS prediction remains a distinct continuous-regression task that imposes application-specific requirements, including clinically meaningful temporal responsiveness, robustness to artifact contamination and inter-patient variability, and evaluation under realistic anaesthesia-monitoring conditions. Despite substantial progress in EEG-based DoA monitoring, several limitations remain. First, many high-performing systems still depend on multichannel EEG, which complicates wearable implementation, increases acquisition and computational burden, and reduces suitability for low-latency bedside deployment. Second, most single-lead approaches continue to rely on hand-crafted descriptors, including entropy-, wavelet-, and variability-based features combined with conventional regressors^20–27^. Although effective in controlled settings, such pipelines are strongly influenced by feature-design choices and often provide limited evidence of robustness across artifacts, inter-patient variability, and differing monitoring conditions. Third, end-to-end deep models that operate directly on EEG waveforms are often difficult to deploy efficiently in clinical environments: large recurrent stacks introduce sequential bottlenecks and tuning sensitivity^28,29^, while transformer-based models are typically data-hungry, computationally expensive, and less attractive for low-latency operation, especially when causal or near-causal inference is desired^19^. In addition, the literature remains difficult to compare directly because studies frequently differ in endpoints, evaluation metrics, and data-partitioning protocols, thereby limiting reproducibility and providing little practical guidance for deployment on modest clinical hardware.

In response to these limitations, the present study develops a lightweight single-channel framework for continuous BIS estimation from raw or minimally processed frontal EEG. The preprocessing stage is designed to preserve EEG–BIS temporal alignment while improving robustness under practical monitoring conditions. At the modeling stage, the framework combines a dilated temporal convolutional network (TCN) for efficient multiscale temporal feature extraction, a compact BiLSTM for within-window contextual refinement, additive attention pooling for adaptive aggregation of temporally informative representations, and a causal EWMA post-processing step to stabilize predictions without using information beyond the current decision window. The study also contributes a comprehensive evaluation protocol that examines the framework from both methodological and deployment perspectives, including architectural ablations, temporal smoothing analysis, agreement assessment, BIS-range error stratification, strict subject-independent 5-fold GroupKFold validation, benchmarking against representative single-lead methods, and computational-efficiency reporting. With approximately 1.07 million parameters and a batched inference throughput of about 430–440 segments per second, the resulting system provides an efficient, interpretable, and practically deployable approach to continuous BIS estimation under single-sensor constraints.

The remainder of the manuscript is structured as follows. Section 2 details the dataset and preprocessing, and presents the proposed TCN–BiLSTM–attention architecture together with the training, inference, and evaluation protocol. Section 3 reports the experimental results, including random segment-level analyses, ablation studies, subject-independent evaluation, and comparisons with prior work. Section 4 discusses implications, limitations, and future directions, and Sect. 5 concludes the paper.

## Methodology

### Dataset description

We analyzed a publicly available perioperative dataset^39^ comprising a single frontal-lead EEG recorded simultaneously with Bispectral Index (BIS) values from 24 adult surgical patients. Cases involved either total intravenous anaesthesia (TIVA) or a combined intravenous–inhalational technique (CIVIA). All participants were ASA Physical Status I–II. The cohort’s mean age was 44.5 ± 12.9 years, mean height 164.2 ± 7.1 cm, mean weight 63.4 ± 14.8 kg, and mean BMI 23.4 ± 4.2 kg/m²; 14 patients were female and 10 were male. Surgeries lasted 126.4 ± 72.9 min on average. Anaesthesia was induced with intravenous propofol, a neuromuscular blocker, and fentanyl, and was maintained with either propofol or an inhalational agent (desflurane or sevoflurane) titrated to 1.0–1.5 MAC. Additional EEG-active medications, such as ketamine and morphine, were administered at the clinician’s discretion. Physiologic monitoring began 5 min before induction and continued until patients responded to verbal commands during emergence. The frontal EEG was sampled at 128 Hz, and BIS values were recorded every 5 s using a BIS Quatro sensor (Aspect Medical Systems, Newton, MA, USA). Vital signs, including non-invasive blood pressure, heart rate, pulse rate, and peripheral oxygen saturation, were also acquired at 5-s intervals and synchronized with the EEG and BIS streams. Additional details regarding the source dataset are provided in^20^.

**Fig. 1:**
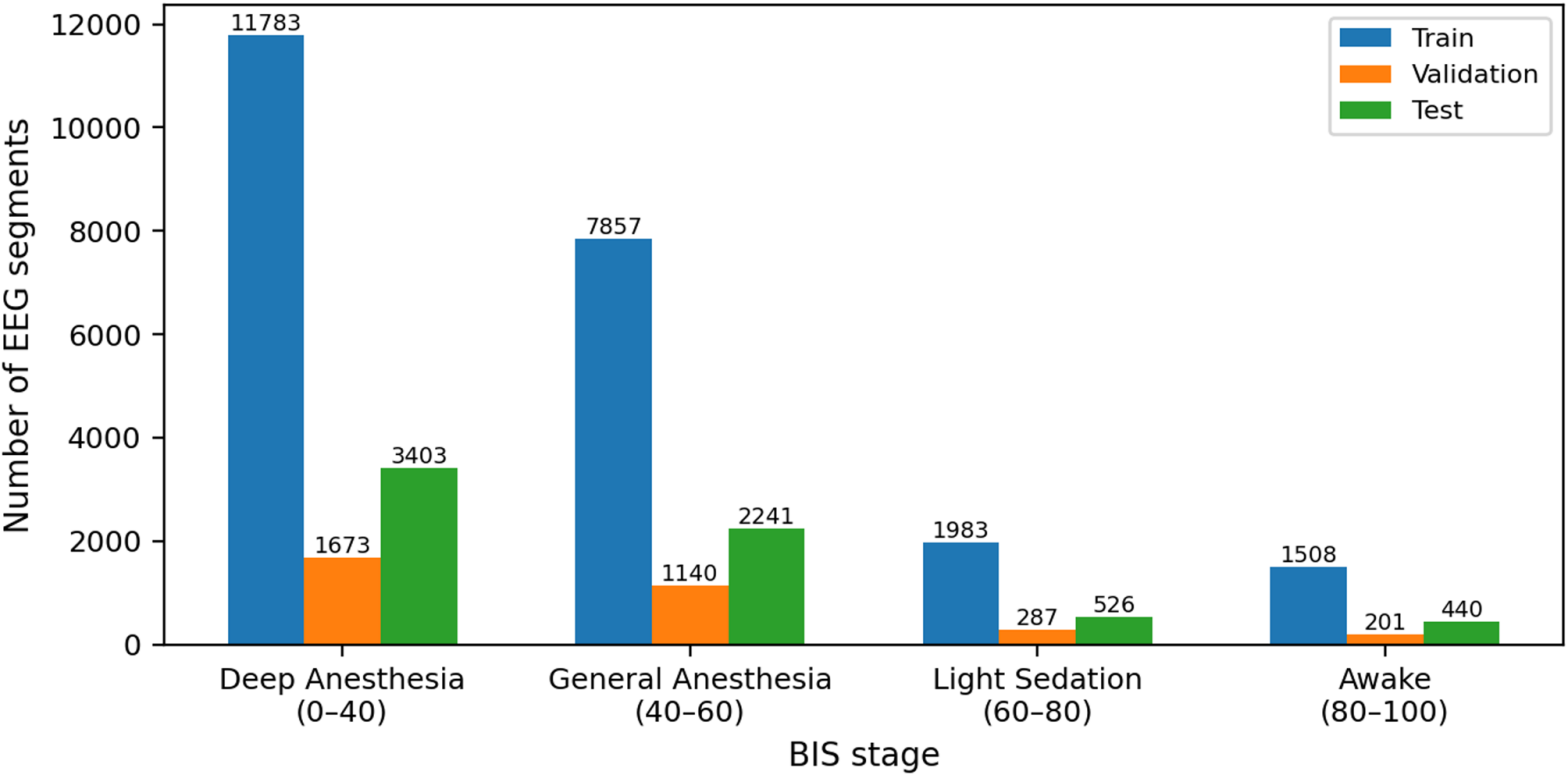
Distribution of EEG segments across BIS stages in the training, validation, and test sets.

The final analysis corpus used in this study comprised 33,435 non-overlapping 5-s EEG segments from the 24 subjects. For clinical interpretability, BIS labels were grouped into four stages: deep anaesthesia (0–40), general anaesthesia (40–60), light sedation (60–80), and awake (80–100). Of the total segments, 393 had BIS values outside the nominal 0–100 range and were excluded from stage-wise distribution analysis, leaving 33,042 valid segments. The resulting dataset was imbalanced across BIS stages: deep anaesthesia, *n* = 16,859 (51.02%); general anaesthesia, *n* = 11,238 (34.01%); light sedation, *n* = 2,796 (8.46%); and awake, *n* = 2,149 (6.50%), as summarized in Table 1. Figure 1 further demonstrates that the BIS-stage distributions were broadly consistent across the training, validation, and test sets. The corresponding segment counts for deep anaesthesia were 11,783, 1,673, and 3,403; for general anaesthesia, 7,857, 1,140, and 2,241; for light sedation, 1,983, 287, and 526; and for the awake state, 1,508, 201, and 440, respectively. Overall, the dataset was predominantly composed of deep and general anaesthesia segments, with comparatively fewer segments representing light sedation and awake states.

**Table 1:** Distribution of EEG segments across BIS stages in the final analysis corpus.

BIS stage	Segments (*n*)	Percentage within 0–100 (%)	Duration (min)	Duration (h)
Deep anaesthesia (0–40)	16,859	51.02	1404.92	23.42
General anaesthesia (40–60)	11,238	34.01	936.5	15.61
Light sedation (60–80)	2,796	8.46	233	3.88
Awake (80–100)	2,149	6.5	179.08	2.98
Outside 0–100	393	—	32.75	0.55

### Preprocessing

**Fig. 2:**
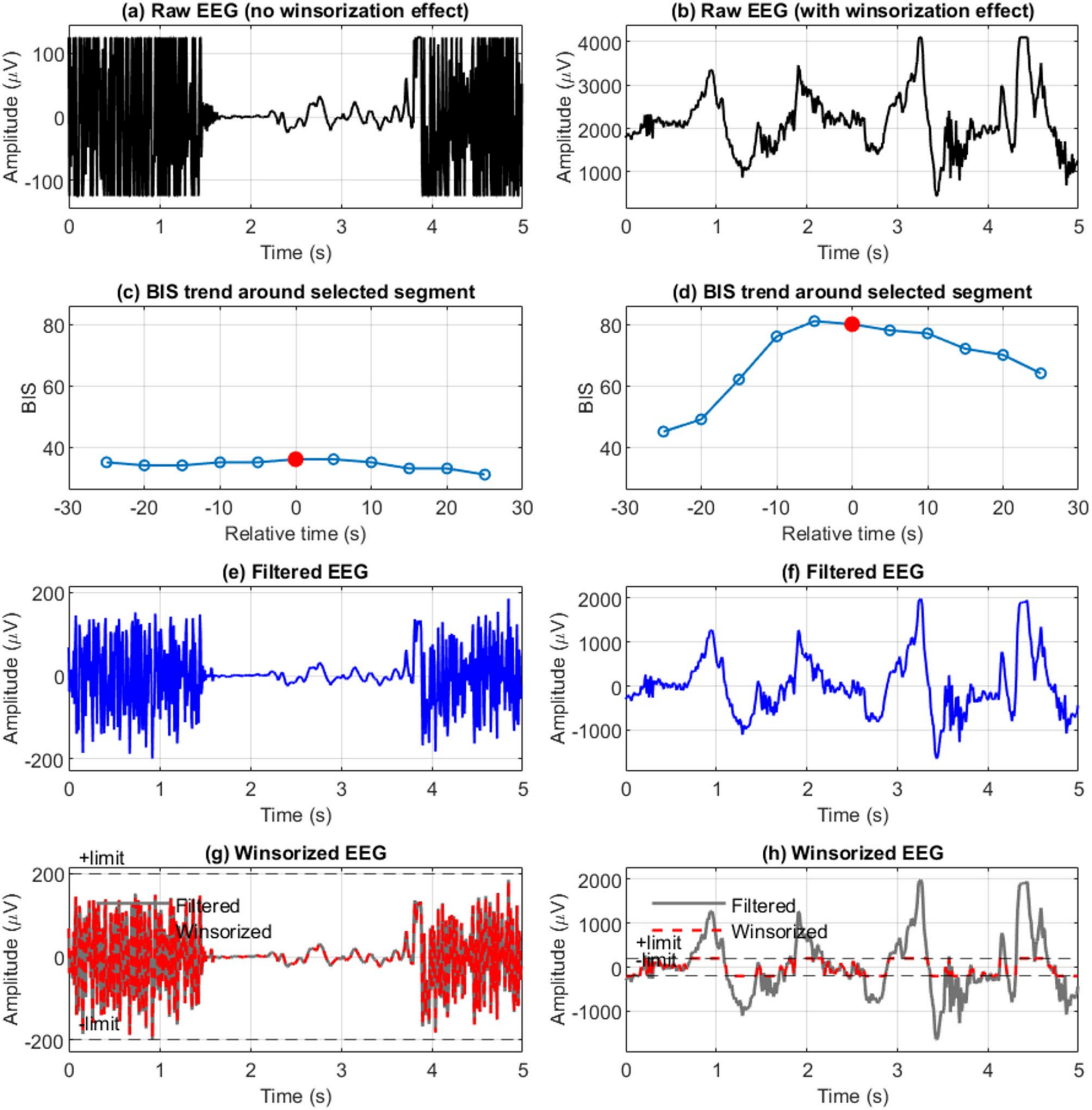
Representative EEG preprocessing examples with corresponding BIS trends. The left column shows a 5-s segment unaffected by winsorization, and the right column shows a 5-s segment in which winsorization clipped brief extreme amplitudes. From top to bottom, panels show raw EEG, local BIS trend, filtered EEG, and winsorized EEG. Red markers in the BIS panels indicate the BIS value corresponding to the displayed EEG segment.

Continuous single-frontal EEG was preprocessed using a lightweight pipeline designed to balance signal cleaning, label fidelity, and compatibility with low-latency BIS estimation. First, the EEG was filtered using a fourth-order zero-phase Butterworth band-pass filter (0.5–50 Hz). The Butterworth design provides a flat passband and moderate roll-off, making it suitable for preserving waveform morphology while suppressing unwanted spectral components. The 0.5 Hz cutoff attenuates slow drift and electrode-related fluctuations, whereas the 50 Hz cutoff retains the main anaesthesia-relevant EEG content while reducing higher-frequency noise, including EMG. While some earlier studies used an upper cutoff of 47 Hz^28,40^, we adopted 50 Hz to preserve slightly broader EEG information^27^ and handled line interference separately using a 60 Hz notch filter. With a sampling rate of 128 Hz, this upper bound also remains safely below the Nyquist frequency. Residual mains interference was further attenuated using an IIR notch filter at 60 Hz^20^(normalized center frequency $\:{\omega\:}_{0}=60/({f}_{s}/2)$, bandwidth $\:{\omega\:}_{0}/35$, quality factor $\:\approx\:35$). The filtered EEG was then segmented into contiguous, non-overlapping 5-s windows (640 samples at 128 Hz). This window length was chosen primarily to match the BIS sampling interval, because BIS measurements were available every 5 s and could therefore be aligned one-to-one with consecutive EEG segments. This design avoids target interpolation, repeated labels across overlapping windows, and aggregation of multiple BIS values into a single training target. In addition, from the perspective of deployment, a 5-s window provides a practical compromise between temporal context and latency within the proposed window-level near-real-time framework. Although longer windows may capture more extended temporal information, they would also increase latency and weaken the direct alignment between each EEG segment and its corresponding BIS label. Incomplete trailing samples shorter than 5 s were discarded to maintain uniform input length. When the EEG and BIS streams differed in length, the longer stream was truncated to match the shorter stream and thereby prevent temporal misalignment between signal and label. Several basic data-quality assurance steps were then applied before model training. Non-finite values were removed before export, and each retained segment was stored together with its scalar BIS label and subject identifier. Rather than applying an aggressive hard-rejection strategy that could discard substantial data or require an external signal-quality index, we adopted a robustness-oriented approach to artifact handling. Specifically, EEG amplitudes were winsorized to $\:\pm\:200\mu\:$V^28^ and then z-scored on a per-subject basis using statistics computed only from that subject’s segments within the corresponding train, validation, or test split. This split-safe normalization prevents cross-split leakage while reducing inter-subject amplitude variability. The $\:\pm\:200\mu\:$V threshold was used as a pragmatic outlier-control step: instead of discarding an entire segment when a small number of samples exhibited large-amplitude excursions, only the extreme sample values were capped. In our dataset, this preprocessing step had a limited effect: only 791 of 33,042 valid segments (2.394%) contained any samples beyond $\:\pm\:200\mu\:$V, and only 65,125 of 21,146,880 total samples (0.308%) were affected. Moreover, the 95th percentile of the per-segment maximum absolute amplitude was 158.99 $\:\mu\:$V, below the winsorization threshold, indicating that clipping primarily affected a small minority of high-amplitude excursions rather than broadly suppressing physiological EEG activity. Representative examples of the preprocessing pipeline, including segments with and without winsorization effect and their corresponding BIS trends, are shown in Fig. 2. We therefore interpret winsorization as a conservative artifact-robustness measure rather than a strong denoising operation, while acknowledging that exceptionally large physiological or artifactual bursts may still be attenuated. Overall, the preprocessing pipeline was intentionally designed to be simple and deployment-compatible: filtering reduced common spectral contaminants, segmentation preserved direct EEG-BIS alignment, stream truncation prevented label mismatch, and winsorization plus split-safe per-subject normalization improved robustness without requiring heavy offline artifact rejection. No separate hard-rejection stage based on a formal EEG signal-quality index was applied; instead, residual artifacts were handled through filtering, conservative amplitude control, normalization, and the downstream temporal weighting capacity of the model. These preprocessing steps produced the final analysis corpus described in Sect. 2.1.

### Proposed TCN-BiLSTM-attention model for BIS prediction

We propose a single-lead EEG framework for continuous BIS estimation from streaming frontal EEG. Intraoperative EEG is inherently noisy and non-stationary, with temporal structure spanning slow drifts, alpha- and beta-band activity, burst-suppression patterns, and brief transient contaminations such as EMG or cautery artifacts. To accommodate these characteristics, the proposed model adopts a TCN–BiLSTM–attention architecture for single-lead BIS regression. Temporal convolutional networks (TCNs), introduced by Bai et al.^41^, provide an efficient sequence-modeling framework based on causal convolutions, dilated receptive fields, and residual connections. These properties enable multiscale temporal modeling while preserving parallelism and maintaining moderate computational cost. TCNs have shown strong performance across diverse sequence-learning tasks, including forecasting^42,43^, classification, and detection^44,45^. Building on these strengths, the present model uses a compact BiLSTM to refine temporal dependencies within each analysis window^38^, followed by additive attention pooling to aggregate the sequence into a saliency-weighted representation for BIS prediction^39^. This combination allows the model to capture both local and broader temporal structure while emphasizing diagnostically informative intervals. Similar design principles have also been effective in related sequence-learning settings^40,41^.

**Fig. 3:**
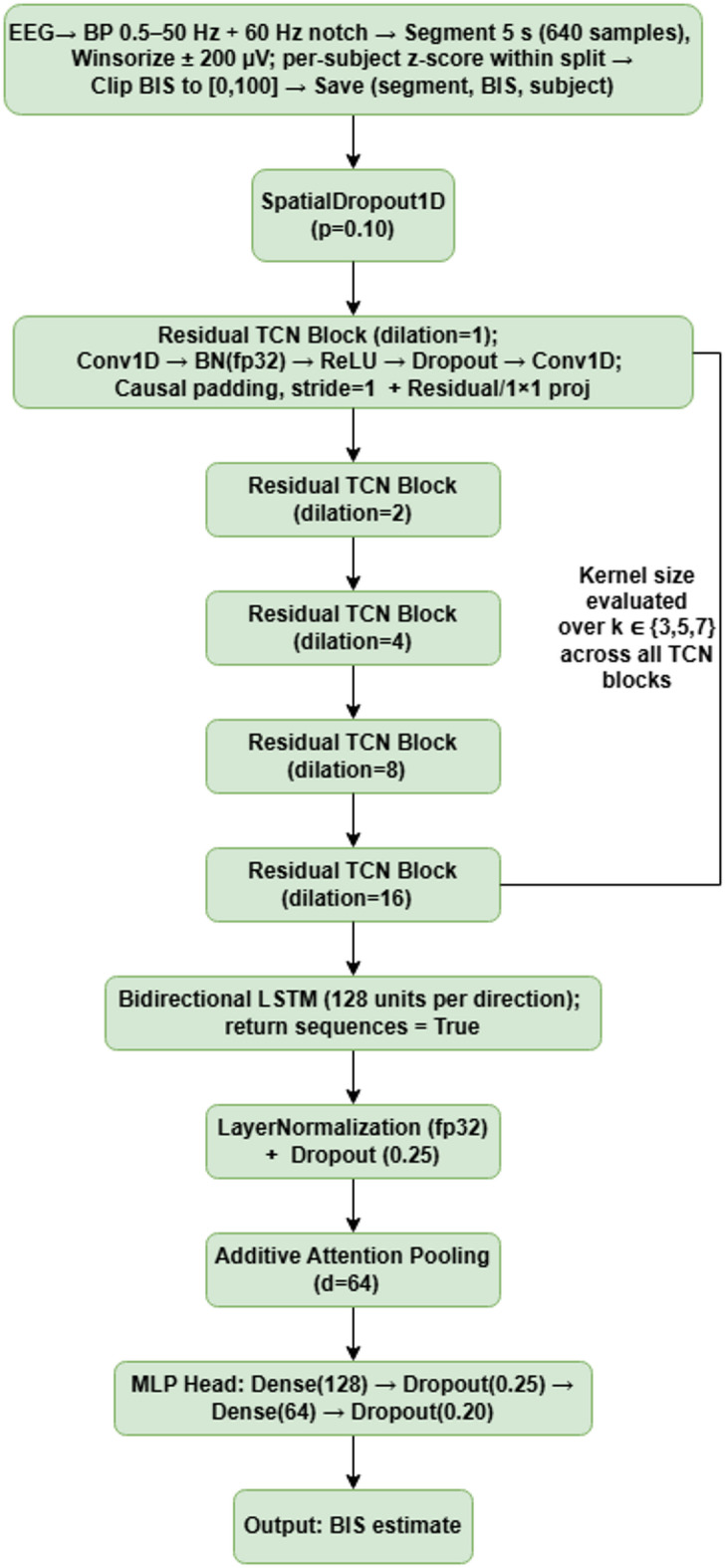
End-to-end pipeline for BIS prediction from single-frontal EEG.

The complete architecture, illustrated in Fig. 3, begins with a causal dilated TCN that extracts multiscale temporal features from the input EEG. The backbone comprises five residual TCN blocks with dilation factors $\:\{\mathrm{1,2},\mathrm{4,8},16\}$, while the kernel size is treated as a tunable architectural hyperparameter and evaluated over $\:k\in\:\{\mathrm{3,5},7\}$. Each block contains two one-dimensional causal convolutions with 128 filters and a stride of 1, each followed by batch normalization and a ReLU activation, with a dropout rate of 0.10 inserted between the two convolutions. Batch normalization is maintained in float32 to ensure numerical stability during mixed-precision training. Residual skip connections support gradient flow and facilitate optimization, and a $\:1\times\:1$ projection is used whenever the input and output channel dimensions differ. With two convolutions per block, the effective receptive field is

1 $$\:R=1+2(k-1)\sum\:_{l=1}^{5}{d}_{l}$$

where $\:{d}_{l}\in\:\{\mathrm{1,2},\mathrm{4,8},16\}$ denotes the dilation factor of the * l*-th residual block. Under the evaluated kernel settings, this corresponds to receptive-field spans of 125, 249, and 373 samples for * k=3*, * k=5*, and * k=7*, respectively, equivalent to approximately 0.98 s, 1.95 s, and 2.91 s at 128 Hz. This design enables a controlled comparison of receptive-field width while keeping the backbone depth and dilation schedule unchanged across model variants.

The resulting TCN feature sequence is then processed by a BiLSTM with 128 hidden units in each direction and configured to return the full sequence. This stage complements the convolutional front end by modeling contextual dependencies across the complete 5-s segment. The recurrent output is regularized with dropout of 0.25 and stabilized using layer normalization in float32. Because the BiLSTM is bidirectional, it uses both earlier and later samples within the current analysis window. Consequently, although the TCN front end is strictly causal, the overall TCN–BiLSTM–attention model is not strictly causal at the sample level. Instead, the system operates in a window-level causal mode: each BIS estimate is produced only after the full trailing 5-s segment has been observed. Thus, the model does not use information beyond the current decision window, while accepting a fixed latency equal to the window length, which remains compatible with low-latency and near-real-time deployment.

To convert the BiLSTM output sequence into a fixed-dimensional representation suitable for regression, we apply additive attention pooling over the sequence of BiLSTM hidden states. Let $\:{H=\left\{{h}_{t}\right\}}_{t=1}^{T}$ denote the sequence of BiLSTM hidden states. For each time step, a small scoring network assigns an unnormalized attention score $\:{e}_{t}$ as given in Eqs. (2),

2 $$\:{e}_{t}={v}^{T}\mathrm{tanh}\left(W{h}_{t}+b\right),\:$$

where * W*, * b*, and * u* are learnable parameters, and $\:\mathrm{tanh}(\cdot\:)$ provides a bounded nonlinearity. These scores are then normalized with a softmax to obtain attention weights $\:{\alpha\:}_{t}$ as in Eq. (3)

3 $$\:{\alpha\:}_{t}=\:\frac{\mathrm{e}\mathrm{x}\mathrm{p}\left({e}_{t}\right)}{\sum\:_{i=1}^{T}\mathrm{e}\mathrm{x}\mathrm{p}\left({e}_{i}\right)},\:\:\sum\:_{t=1}^{T}{\alpha\:}_{t}=1,\:\:\:{\alpha\:}_{t}\ge\:0,$$

The pooled context vector * c* given by Eq. (4) is the convex combination

4 $$\:c=\sum\:_{t=1}^{T}{\alpha\:}_{t}\:{h}_{t}$$

In this way, the model learns to assign greater importance to informative time steps while reducing the influence of noisy or artifact-dominated regions. In the proposed architecture, the attention hidden size is 64, providing a compact yet expressive scoring function for temporal weighting. For numerical stability, the softmax is implemented in shifted form, and padded positions, if any, are masked before normalization. Compared with transformer self-attention, which models all pairwise temporal interactions at quadratic cost, this additive attention mechanism scales linearly with sequence length and is therefore more suitable for the intended lightweight deployment setting. Beyond improving temporal aggregation for BIS prediction, the resulting attention weights also support post hoc interpretability by indicating which parts of the EEG segment contribute most strongly to the final estimate. Detailed analyses of these learned attention patterns are presented in Sect. 3.1.6.

The attention-pooled vector is further mapped to a scalar BIS estimate by a compact multilayer perceptron consisting of a dense layer of width 128 followed by dropout of 0.25, a second dense layer of width 64 followed by dropout of 0.20, and a final linear output unit. No hard bounds are imposed during training; predictions are clipped to the physiologically valid range of 0 to 100 only for reporting. For clarity, the complete block-wise configuration of the proposed TCN-BiLSTM-attention architecture is summarized in Table 2.

**Table 2:** Block-wise configuration of the proposed TCN-BiLSTM-attention model.

Stage	Layer/block	Configuration	Output role
Input	EEG segment	Single-channel 5-s segment sampled at 128 Hz	Raw temporal input
Feature extractor	Residual TCN Block 1	2 × causal Conv1D, 128 filters, kernel size $\:k\in\:\{\mathrm{3,5},7\}$, dilation 1, batch normalization, ReLU, dropout 0.10, residual skip	Local temporal feature extraction
Feature extractor	Residual TCN Block 2	2 × causal Conv1D, 128 filters, kernel size $\:k\in\:\{\mathrm{3,5},7\}$, dilation 2, batch normalization, ReLU, dropout 0.10, residual skip	Expanded temporal context
Feature extractor	Residual TCN Block 3	2 × causal Conv1D, 128 filters, kernel size $\:k\in\:\{\mathrm{3,5},7\}$, dilation 4, batch normalization, ReLU, dropout 0.10, residual skip	Multi-scale feature extraction
Feature extractor	Residual TCN Block 4	2 × causal Conv1D, 128 filters, kernel size $\:k\in\:\{\mathrm{3,5},7\}$, dilation 8, batch normalization, ReLU, dropout 0.10, residual skip	Longer-range temporal context
Feature extractor	Residual TCN Block 5	2 × causal Conv1D, 128 filters, kernel size $\:k\in\:\{\mathrm{3,5},7\}$, dilation 16, batch normalization, ReLU, dropout 0.10, residual skip	Highest TCN temporal coverage
Sequence refinement	BiLSTM	128 units per direction, return sequences, layer normalization, dropout 0.25	Bidirectional within-window temporal modeling
Temporal aggregation	Additive attention	Hidden size 64; attention scoring, softmax normalization, and weighted temporal pooling	Saliency-weighted sequence summary
Regression head	Dense 1	128 units + dropout 0.25	Nonlinear feature transformation
Regression head	Dense 2	64 units + dropout 0.20	Compact regression refinement
Output	Linear layer	1 unit	Scalar BIS prediction

The full network is trained end-to-end using the Huber loss with $\:\delta\:=5$, optimized with AdamW using weight decay $\:1\times\:{10}^{-4}$ and gradient clip-norm 1.0. The learning rate follows a warm-up cosine schedule with three warm-up epochs, a total of 120 epochs, a base learning rate of $\:1\times\:{10}^{-3}$, and a minimum learning rate of $\:1\times\:{10}^{-6}$. Training is performed with a batch size of 64 and mixed precision enabled. Early stopping is applied based on validation loss with patience 18 and restoration of the best checkpoint, while the validation and test pipelines are kept deterministic and only the training data are shuffled.

The architecture was designed to balance temporal coverage, representational capacity, and robustness for single-channel EEG segments. Consistent with the preprocessing design, the model operates on 5-s windows aligned to the BIS update interval. Within this setting, the TCN kernel size and dilation schedule were chosen to capture local and mid-range waveform structure without excessive depth or parameter growth, while the BiLSTM size was selected to provide sufficient sequence-modeling capacity without making the recurrent stage disproportionately large relative to the dataset size and single-channel input. The attention hidden size was kept compact to enable temporally selective weighting of informative hidden states with limited parameter overhead. Overall, the final hyperparameters were determined through iterative, validation-guided tuning, with emphasis on validation MAE, training stability, and parameter efficiency. The rationale for the main architectural and hyperparameter choices is summarized in Table 3.

**Table 3:** Rationale for major architectural and hyperparameter choices.

Component	Setting	Rationale
Input window length	5 s	Matches the BIS update interval and enables direct one-to-one supervision between each EEG segment and its BIS label
TCN kernel size	$\:k\epsilon\{\mathrm{3,5},7\}$	Evaluated to examine the effect of receptive-field width on BIS prediction while keeping the backbone depth and dilation schedule unchanged
TCN dilations	1, 2, 4, 8, 16	Expands receptive field efficiently for multi-scale temporal modeling without excessive depth
Number of TCN blocks	5	Balances temporal coverage and model depth for single-channel EEG
Convolutions per TCN block	2	Improves representational capacity while maintaining a manageable parameter count
TCN filters	128	Provides sufficient feature capacity for single-channel input without over-expanding the convolutional stage
TCN receptive field	125, 249, and 373 samples for (k = 3,5,7), respectively (≈ 0.98 s, 1.95 s, and 2.91 s at 128 Hz)	Provides a controlled range of local-to-mid temporal coverage across the evaluated kernel settings while maintaining computational efficiency
Residual connections	Yes	Preserve gradient flow and stabilize optimization in deeper dilated convolutional stacks
TCN dropout	0.1	Adds regularization with limited disruption to temporal feature extraction
BiLSTM units	128 per direction	Provides adequate sequence-modeling capacity across the 5-s window without making the recurrent layer disproportionately large
BiLSTM output mode	Return sequences	Required so attention pooling can assign temporal importance weights over the full sequence
BiLSTM dropout	0.25	Regularizes the recurrent stage and helps reduce overfitting
Attention hidden size	64	Enables compact scoring of temporal importance while limiting parameter growth
Regression head	128 → 64 → 1	Provides a lightweight regression head after temporal aggregation
Loss function	Huber loss $\:(\delta\:=5)$	Improves robustness to outliers while retaining sensitivity to moderate prediction errors
Optimizer	AdamW	Provides stable optimization with decoupled weight decay
Weight decay	$\:1\times\:{10}^{-4}$	Regularizes model weights without overly constraining learning
Gradient clip-norm	1	Helps stabilize training, particularly in the recurrent stage
Learning-rate schedule	Warm-up cosine	Supports stable early training and gradual decay toward convergence
Batch size	64	Balances training stability, throughput, and memory usage
Early stopping	Patience 18	Reduces overfitting by restoring the best validation checkpoint

### Evaluation metrics

We assessed BIS prediction accuracy using mean absolute error (MAE), root-mean-squared error (RMSE), and the coefficient of determination (R²) as specified in Eqs. (5)-(7). MAE and RMSE (in BIS units) quantify average error magnitude and dispersion, respectively (lower is better), whereas R² indicates goodness of fit, with values closer to 1 reflecting a greater proportion of BIS variance explained by the model. We also report Pearson’s correlation coefficient (* r*) given in Eq. (8) to measure linear association and Lin’s concordance correlation coefficient (CCC) provided in Eq. (9) to assess agreement by jointly reflecting correlation and calibration; values closer to 1 indicate stronger agreement.

5 $$\:MAE=\frac{1}{N}\:(\:{{\sum\:}_{\left\{i=1\right\}}^{N}|y}_{i}-\:\widehat{{y}_{i}}|\:)$$

6 $$\:RMSE=\sqrt{\frac{1}{N}{\sum\:}_{\left\{i-1\right\}}^{N}({\:{y}_{i}-\:\widehat{{y}_{i}})}^{2}}$$

7 $$\:{R}^{2}=1-\frac{{\sum\:}_{\left\{i-1\right\}}^{N}({\:{y}_{i}-\:\widehat{{y}_{i}})}^{2}}{{\sum\:}_{\left\{i-1\right\}}^{N}({\:{y}_{i}-\:\stackrel{-}{{y}_{i}})}^{2}}$$

8 $$\:Pearson,r=\frac{{\sum\:}_{\{i=1\}}^{N}({y}_{i}\:-\:\stackrel{-}{y})({\widehat{y}}_{i}-\:\stackrel{-}{\widehat{y}})}{\sqrt{\sum\:_{\{i=1\}}^{N}{({y}_{i}\:-\:\stackrel{-}{y})}^{2}}\sqrt{\sum\:_{i=1}^{N}{({\widehat{y}}_{i}-\:\stackrel{-}{\widehat{y}})}^{2}}}$$

9 $$\:Li{n}^{{\prime\:}}s\:CCC=\frac{2\rho\:{\sigma\:}_{y}{\sigma\:}_{\widehat{y}}}{{\sigma\:}_{y}^{2}+\:{\sigma\:}_{\widehat{y}}^{2}+\:{(\stackrel{-}{y}-\:\stackrel{-}{\widehat{y}})}^{2}},\:\:\rho\:=r$$

where $\:{y}_{i}$, $\:\widehat{{y}_{i}}$, $\:\stackrel{-}{{y}_{i}}$ respectively represent the actual value, predicted value, and the mean of actual values; * N* denotes the data quantity. All evaluation metrics are reported under two conditions: (i) RAW, corresponding to the instantaneous model outputs, and (ii) EWMA, corresponding to a per-subject causal exponentially weighted moving average applied to the RAW predictions. EWMA is included in the proposed framework as a temporal stabilization component intended to reduce short-term variability and produce smoother BIS-aligned trajectories. Because the reported BIS target is itself a processed and temporally smoothed monitor output, this additional smoothing can improve agreement with the target trajectory. However, it also introduces an inherent trade-off: although EWMA improves temporal stability, it may reduce responsiveness to abrupt anesthetic state transitions relative to the RAW model output. Accordingly, the EWMA predictions should be interpreted primarily as stabilized BIS-aligned trend estimates, whereas the RAW predictions more directly reflect the model’s immediate temporal response. For completeness and transparency, both forms of output are reported in the present study. The EWMA smoothing parameter $\:\alpha\:$ was selected by grid search over the range 0.05 to 0.35 in increments of 0.05. For each candidate value, EWMA smoothing was applied in a causal, subject-wise manner to the validation predictions, and the corresponding MAE was computed. The value of $\:\alpha\:$ that minimized validation MAE was then fixed and used for all test-set evaluations. This procedure ensures that the smoothing parameter is selected independently of the test data while providing a practical balance between temporal stability and responsiveness of the BIS estimates.

### Statistical analysis

To assess whether differences among temporal convolution kernel sizes were statistically significant, models with kernel sizes * k=3*, * k=5*, and * k=7*were compared using per-subject test-set MAE as the primary inferential measure. Per-subject MAE was preferred over pooled segment-level error so that subjects contributing larger numbers of EEG segments did not dominate the comparison and the analysis more directly reflected patient-level performance. Because the same held-out subjects were evaluated under each kernel setting, paired nonparametric tests were used. An omnibus Friedman test was first applied to assess whether overall differences existed among the three kernel sizes. Pairwise comparisons were then performed using Wilcoxon signed-rank tests, with Holm correction applied across the three pairwise contrasts: * k=3* vs. * k=5*, * k=3* vs. * k=7*, and $\:k=5\:$vs. * k=7*. These analyses were conducted separately for the random segment-level and subject-independent evaluation settings, and separately for RAW and EWMA-smoothed predictions. In the random segment-level setting, per-subject MAE was computed from the held-out test split for each kernel. In the subject-independent setting, per-subject MAE was computed from pooled out-of-fold test predictions across the 5-fold GroupKFold protocol, such that each subject contributed one pooled out-of-fold MAE value per kernel. To quantify the effect of temporal post-processing, RAW and EWMA-smoothed predictions were additionally compared for each kernel using paired Wilcoxon signed-rank tests on per-subject MAE, with Holm correction applied across the three kernel-wise comparisons.

## Results and discussions

This section reports a quantitative evaluation of the proposed BIS estimation framework under two experimental protocols. First, we use a random segment-level split with per-subject stratification, where EEG segments are randomly assigned to training, validation, and test sets while preserving each subject’s proportion across splits. This setting supports detailed analysis of model behavior, including temporal smoothing, agreement, and ablation studies. Second, we use a subject-independent protocol based on 5-fold GroupKFold cross-validation, where all segments from a subject are assigned exclusively to a single fold, providing a more deployment-relevant assessment of generalization to unseen patients. Performance is reported using MAE, RMSE, $\:{R}^{2}$, Pearson’s * r*, and Lin’s CCC. Experiments were run on a single Google Colab GPU using mixed-precision training, with batch-normalization layers maintained in float32; random seeds were fixed to 42 for NumPy, TensorFlow, and data shuffling to ensure reproducibility.

### Random segment-level evaluation

Results in this subsection are based on a segment-level split with per-subject stratification, in which segments from each subject are distributed across training, validation, and test sets in fixed proportions. Because segments from the same subject may appear in multiple splits, this protocol is used primarily for diagnostic analysis of architectural choices, smoothing behavior, and error characteristics. Accordingly, the EWMA α-sensitivity analysis, agreement plots, BIS-range stratification, and ablation studies reported in Sect. 3.1.1–3.1.6 are derived from this segment-level setting.

#### EWMA smoothing and α sensitivity

**Fig. 4:**
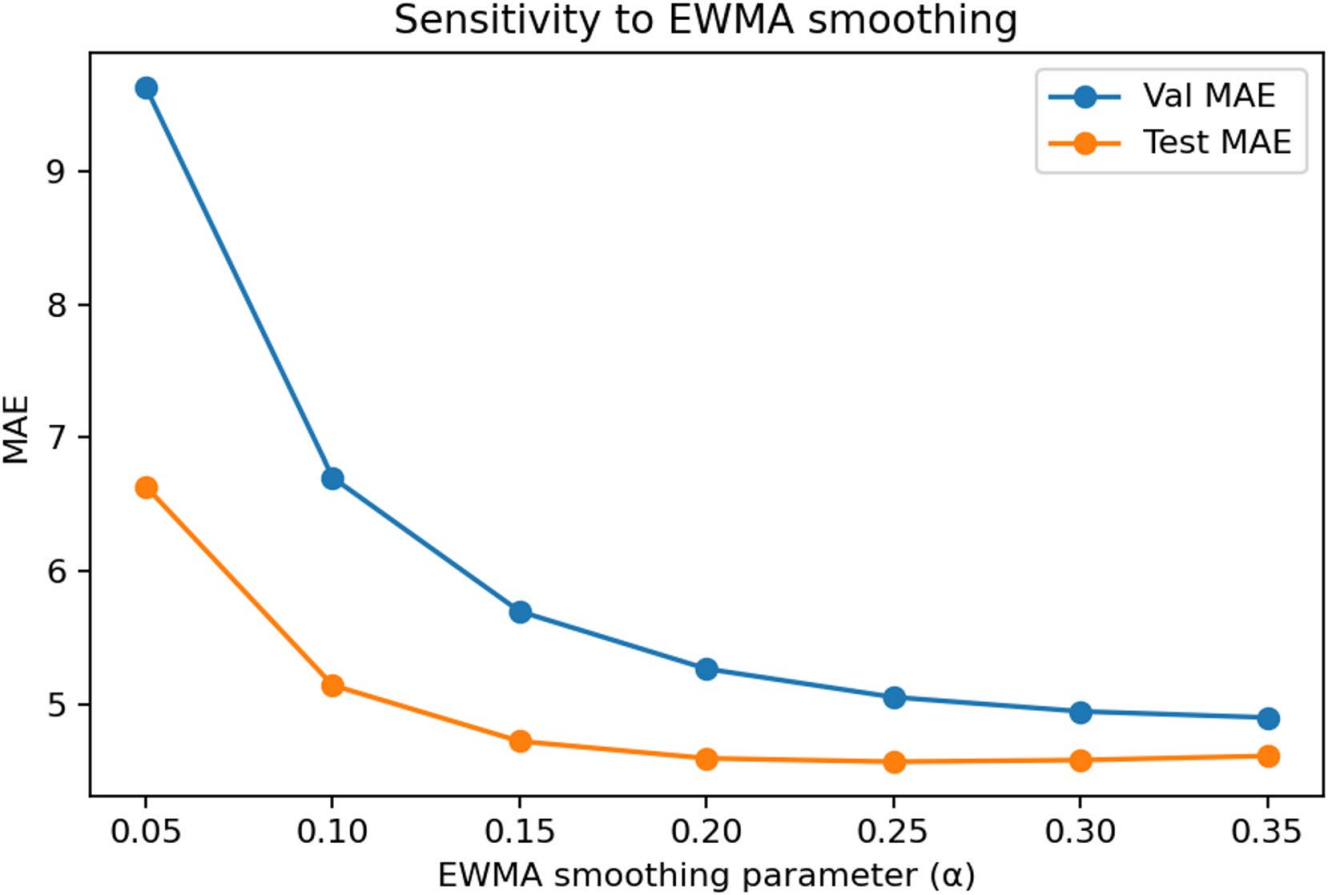
Sensitivity of validation MAE to the EWMA smoothing parameter α.

The smoothing factor $\:\alpha\:$ was selected from 0.05 to 0.35 (step 0.05) by minimizing validation MAE, and was fixed to $\:\alpha\:=0.35$ for all test evaluations in the segment-level setting. Validation performance was relatively stable for $\:\alpha\:\in\:[0.25,\:0.35]$, with $\:\alpha\:=0.35$ yielding the lowest validation error; smaller $\:\alpha\:$ produced overly sluggish estimates, whereas larger values reduced smoothing. The sensitivity analysis is shown in Fig. 4.

#### Main quantitative results

Table 4 reports results for power-of-two–dilated TCNs with * k=3*, * k=5*, and * k=7* under RAW outputs and smoothed outputs using EWMA with $\:\alpha\:=0.35$ selected on validation and fixed for testing. Across all kernels and both validation and test sets, EWMA reduced error and strengthened agreement. On validation, MAE decreased by about 13–15% and RMSE by about 13–16% for every * k*; for example, with * k=3*, MAE fell from 5.307 to 4.534 and RMSE from 8.865 to 7.443, accompanied by gains in $\:{R}^{2}$, Pearson’s * r*, and CCC. The held-out test set showed the same pattern, with * k=5*, MAE declined from 5.356 to 4.499 and RMSE from 9.012 to 7.499, with corresponding increases in $\:{R}^{2}$, * r*, and CCC. Among models, * k=3*+EWMA performed best on validation, whereas * k=5*+EWMA generalized best on test; * k=7*+EWMA ranked between these two. Consequently, in the random segment-level evaluation, k = 5 produced the best aggregate held-out test performance, suggesting that a mid-size kernel was the most suitable operating point in this setting. One possible explanation is that k = 5 provide a more balanced receptive field under the adopted dilation schedule: k = 3 may yield insufficient temporal coverage for slower anesthesia-related EEG dynamics, whereas k = 7 may trade temporal precision for broader context. In contrast, k = 5 appears to preserve a more favorable balance between contextual coverage and local feature resolution. This interpretation is further supported by the surrounding architecture, where the TCN extracts multiscale temporal patterns, the BiLSTM models dependencies within each 5-s window, and EWMA smoothing improves output stability. Accordingly, we interpret k = 5 as the kernel yielding the strongest aggregate segment-level generalization in the random split, while recognizing that this result should be understood within the diagnostic evaluation setting.

**Table 4:** Performance on validation and test sets for RAW outputs and EWMA-smoothed predictions (α = 0.35).

Evaluation metrics	k=3				k=5				k=7			
	Validation		Test		Validation		Test		Validation		Test	
	RAW	EWMA	RAW	EWMA	RAW	EWMA	RAW	EWMA	RAW	EWMA	RAW	EWMA
MAE	5.307	4.534	5.551	4.865	5.474	4.784	5.356	4.499	5.56	4.812	5.502	4.892
RMSE	8.865	7.443	9.228	8.008	9.217	8.017	9.012	7.499	9.204	7.727	9.171	7.942
R²	0.741	0.818	0.712	0.783	0.712	0.782	0.732	0.815	0.721	0.803	0.715	0.787
Pearson’s r	0.862	0.905	0.846	0.887	0.846	0.886	0.858	0.903	0.856	0.899	0.85	0.887
CCC	0.856	0.896	0.839	0.876	0.841	0.878	0.855	0.897	0.851	0.893	0.847	0.882

#### Agreement and error analysis

**Fig. 5:**
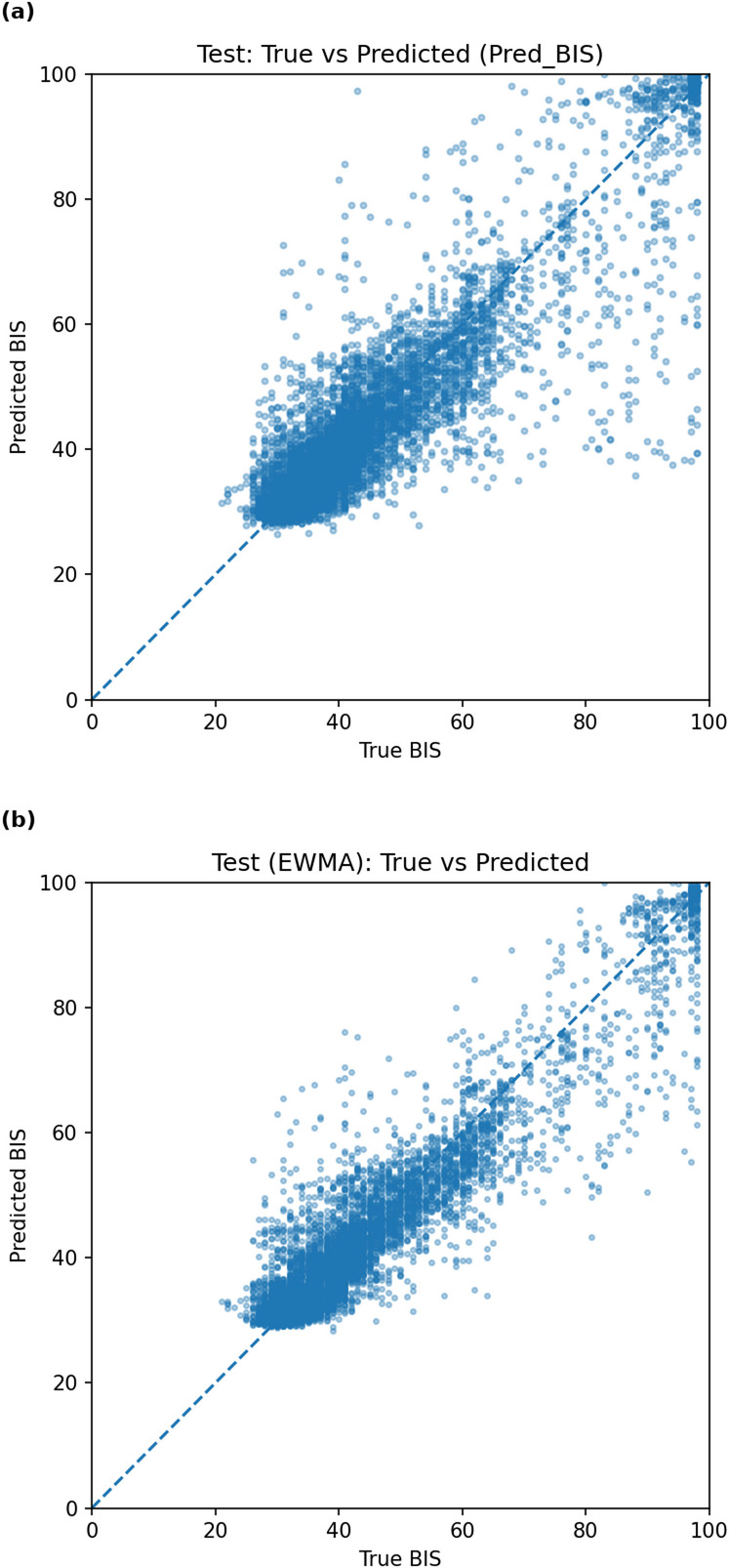
Scatter on test comparing true and predicted BIS with a dashed identity line, showing (**a**) RAW and (**b**) EWMA (α = 0.35).

**Fig. 6:**
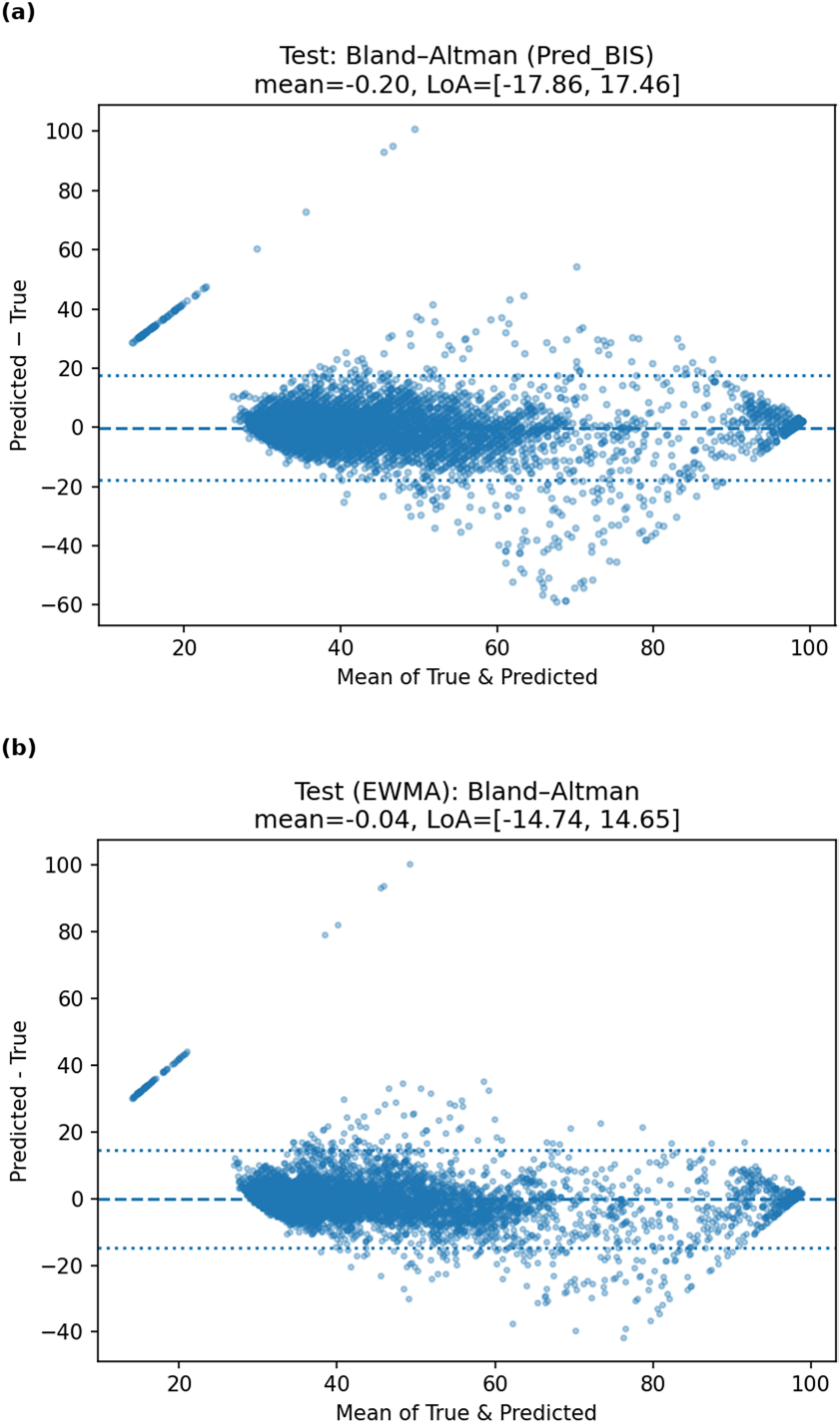
Bland-Altman on test plotting Predicted -True versus the mean with dashed bias and dotted 95% limits of agreement, showing (**a**) RAW and (**b**) EWMA (α = 0.35).

The test-set scatter plots in Fig. 5(a) for RAW predictions and Fig. 5(b) for EWMA-smoothed predictions $\:\left(\alpha\:=0.35\right)$ show a strong monotonic relationship with the identity line. Relative to the RAW outputs, EWMA produces a tighter distribution and fewer extreme residuals across the main operating range of approximately 30–80 BIS. At very high BIS values * (>90)*, both conditions show mild underestimation, whereas at low BIS values * (<30)* the compressed label distribution limits the effective dynamic range. Overall, these scatter plots indicate that EWMA reduces dispersion across the clinically relevant mid-range. While Fig. 5 summarizes the association between predicted and reference BIS values, the Bland-Altman plots in Fig. 6(a) and Fig. 6(b) are more informative for assessing agreement and practical interpretability. Figure 6(a) shows the Bland-Altman analysis for the RAW predictions, with negligible mean bias but relatively wide 95% limits of agreement $\:\left[-\mathrm{17.86,17.46}\right]$, spanning approximately 35 BIS points. This spread is wide enough to cross clinically relevant BIS intervals and could therefore misclassify a patient’s hypnotic state if individual pointwise predictions were interpreted in isolation. By comparison, Fig. 6(b) shows that applying causal EWMA smoothing reduces this dispersion, yielding narrower limits of agreement $\:\left[-\mathrm{14.74,14.65}\right]$ and a greater concentration of observations around zero difference, particularly in the mid-range of approximately 40–70 BIS, where anaesthetic titration is often most critical. These findings suggest that smoothing improves temporal stability and reduces heteroscedasticity; however, the remaining spread is still clinically non-negligible. Accordingly, the proposed framework is better interpreted as a low-latency trend-estimation and decision-support tool rather than as a point-by-point standalone replacement for monitor-derived BIS values. In practical terms, the smoothed trajectories are likely to be more clinically informative than isolated RAW predictions, especially near decision thresholds where even moderate deviations may affect interpretation.

#### Performance across BIS ranges

**Fig. 7:**
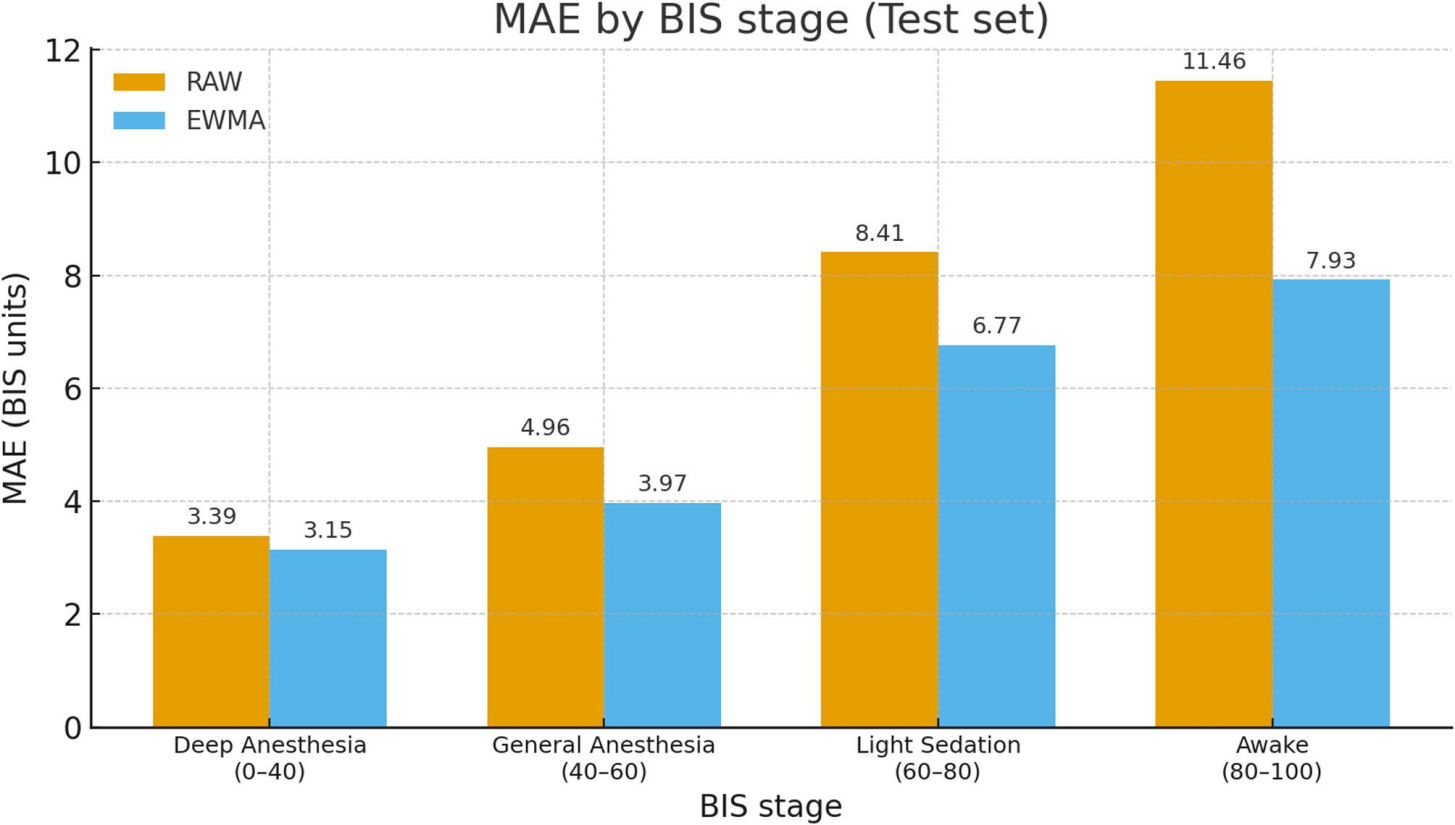
Test-set MAE by BIS stage (RAW vs. EWMA, α = 0.35).

Figure 7 presents test-set MAE stratified by clinical BIS stages. Performance was strongest in moderate BIS ranges. For BIS 20–40, MAE was 3.15 with EWMA versus 3.39 with RAW; for 40–60, MAE was 3.97 versus 4.96. Error increased at higher BIS levels, with MAE values of 6.77 versus 8.41 for 60–80 and 7.93 versus 11.46 for 80–100. No test samples fell within the 0–20 range. Across all populated bins, EWMA consistently reduced error, with larger absolute improvements at higher BIS levels, likely reflecting increased volatility and electromyographic contamination during lighter anaesthesia or emergence.

#### Ablation studies

We conducted a series of ablation studies to isolate the contribution of individual design choices and architectural components under the random segment-level evaluation setting. In each ablation, only a single element of the model or training configuration was modified, while all other factors, including data splits, preprocessing, optimization, and evaluation, were held constant. A full summary of results is provided in Table 5. First, we examined the impact of temporal context modeling by varying the TCN receptive-field design through different dilation schedules. The baseline random segment-level configuration employed power-of-two dilations $\:\left(1,2,4,8,16\right)$and, after EWMA post-processing, achieved MAE = 4.499, RMSE = 7.499, $\:{R}^{2}=0.815$, * r=0.903*, and CCC = 0.897. Replacing this with a linear dilation schedule $\:\left(1\mathrm{-}5\right)$degraded performance (MAE = 5.026, RMSE = 8.103, CCC = 0.870), suggesting reduced ability to capture longer-range temporal dependencies. A geometric schedule $\:\left(1,3,9,27,81\right)$ performed slightly better than the linear alternative but still underperformed relative to the baseline (MAE = 4.899, RMSE = 7.960, CCC = 0.873). In both cases, reductions in $\:{R}^{2}$and CCC suggest that power-of-two dilations provide a favorable balance between rapidly expanding temporal coverage and maintaining stable optimization in this evaluation setting.

We next assessed the effect of temporal depth and the contribution of the recurrent and attention components. Reducing the depth of the TCN $\:\left(\mathrm{dilations\:}[\mathrm{1,2},\mathrm{4,8}]\right)$increased error (MAE = 4.717) and reduced agreement (CCC = 0.888), suggesting the importance of sufficient temporal depth. Replacing the BiLSTM with a unidirectional LSTM led to a smaller but consistent decline in performance (MAE = 4.616, RMSE = 7.748, CCC = 0.893), reflecting the benefit of bidirectional context aggregation within the 5-s analysis window. Substituting additive attention with global average pooling further reduced performance (MAE = 4.654, RMSE = 7.668, CCC = 0.887), indicating that temporally selective aggregation provides an advantage over uniform pooling. Removing recurrence entirely resulted in larger degradations. A TCN-only model with global average pooling yielded MAE = 4.874 and CCC = 0.878, whereas a TCN-only variant with additive attention achieved MAE = 4.968 and CCC = 0.875. These results suggest that, under the random segment-level split, even a lightweight recurrent stage contributes additional temporal refinement beyond what can be captured by convolutional features and pooling alone. Finally, we evaluated design choices regarding optimization stability and computational efficiency. Replacing the Huber loss with MAE or MSE consistently worsened performance (MAE = 4.640, RMSE = 7.714, CCC = 0.889 for MAE; MAE = 4.803, RMSE = 7.652, CCC = 0.887 for MSE). Using split-fit normalization, in which normalization statistics were computed separately within each split, was slightly inferior to the baseline normalization strategy (MAE = 4.695, RMSE = 7.590, CCC = 0.889). The largest performance drop occurred when replacing dilated convolutions with strided temporal convolutions (MAE = 4.822, RMSE = 7.929, CCC = 0.881), suggesting that dilation-based receptive-field expansion preserves temporal detail more effectively than downsampling via stride.

Overall, the ablation results suggest that the observed performance gains arise from the complementary contribution of the major architectural components, rather than from any single module in isolation. In particular, the TCN receptive-field design supports multiscale temporal coverage, sufficient TCN depth improves longer-range temporal modeling, the BiLSTM provides within-window contextual refinement, and the additive attention module improves temporally selective aggregation. The targeted degradation observed under each ablation therefore helps clarify why this particular combination of components was retained in the final framework. Consistent changes in MAE, RMSE, $\:{R}^{2}$, Pearson’s * r*, and CCC across these variants are summarized in Table 5.

**Table 5:** Ablation study results showing the impact of architectural and training variations on validation and test performance under RAW and EWMA post-processing.

Ablation	Post processing	Test set					Validation set				
		MAE	RMSE	*R* ^2^	*r*	CCC	MAE	RMSE	*R* ^2^	*r*	CCC
TCN linear dilation (k = 3)	RAW	5.5109	9.0639	0.7294	0.8558	0.8486	5.6339	9.2576	0.7099	0.8428	0.8332
TCN linear dilation (k = 3)	EWMA	4.7863	7.6357	0.8080	0.9004	0.8903	5.0257	8.1034	0.7778	0.8829	0.8700
TCN linear dilation (k = 5)	RAW	5.5340	9.1125	0.7265	0.8549	0.8457	5.6069	9.1736	0.7152	0.8460	0.8356
TCN linear dilation (k = 5)	EWMA	4.8468	7.6818	0.8056	0.9004	0.8878	5.0344	8.0288	0.7818	0.8857	0.8714
TCN geometric dilation (k = 3)	RAW	5.3779	8.8631	0.7413	0.8618	0.8527	5.5394	9.0922	0.7202	0.8487	0.8375
TCN geometric dilation (k = 3)	EWMA	4.6291	7.5547	0.8120	0.9034	0.8906	4.8988	7.9596	0.7856	0.8887	0.8729
TCN geometric dilation (k = 5)	RAW	5.3666	8.8481	0.7421	0.8622	0.8493	5.4728	8.9749	0.7274	0.8532	0.8387
TCN geometric dilation (k = 5)	EWMA	4.6825	7.6230	0.8086	0.9031	0.8859	4.8773	7.9644	0.7853	0.8897	0.8711
Shallow Depth [1,2,4,8]	RAW	5.4238	9.0907	0.7278	0.8547	0.8491	5.5235	9.1491	0.7167	0.8482	0.8421
Shallow Depth [1,2,4,8]	EWMA	4.7165	7.7559	0.8019	0.8965	0.8877	4.8998	8.0021	0.7833	0.8865	0.8761
LSTM unidirectional	RAW	5.3888	9.1245	0.7258	0.8569	0.8552	5.4068	9.0820	0.7208	0.8538	0.8520
LSTM unidirectional	EWMA	4.6156	7.7480	0.8023	0.8967	0.8928	4.7890	8.1120	0.7773	0.8833	0.8792
POOL_GAP instead of Attention	RAW	5.3428	8.9666	0.7352	0.8578	0.8487	5.4757	9.0996	0.7198	0.8488	0.8386
POOL_GAP instead of Attention	EWMA	4.6537	7.6676	0.8064	0.8998	0.8870	4.8607	7.9838	0.7843	0.8869	0.8734
TCN-only + GAP	RAW	5.5779	9.0679	0.7292	0.8543	0.841	5.6392	9.121	0.7184	0.8483	0.8322
TCN-only + GAP	EWMA	4.8739	7.849	0.7971	0.8967	0.8781	5.0578	8.1485	0.7753	0.8851	0.8637
TCN-only + Additive Attention	RAW	5.7543	9.3052	0.7148	0.8456	0.834	5.7937	9.3089	0.7067	0.8413	0.8283
TCN-only + Additive Attention	EWMA	4.9677	7.9704	0.7908	0.8923	0.8749	5.1494	8.2311	0.7707	0.8816	0.8625
MAE loss	RAW	5.4240	9.0605	0.7296	0.8553	0.8500	5.4882	9.0666	0.7218	0.8508	0.8450
MAE loss	EWMA	4.6399	7.7137	0.8040	0.8974	0.8887	4.7914	7.8636	0.7907	0.8902	0.8808
MSE loss	RAW	5.4530	8.7877	0.7457	0.8635	0.8537	5.5144	8.8565	0.7345	0.8571	0.8466
MSE loss	EWMA	4.8031	7.6519	0.8072	0.9005	0.8869	4.9677	8.0084	0.7829	0.8862	0.8726
NORM_splitfit	RAW	5.3779	8.8487	0.7421	0.8620	0.8520	5.5658	9.0868	0.7206	0.8490	0.8366
NORM_splitfit	EWMA	4.6946	7.5896	0.8103	0.9027	0.8887	4.9560	8.0691	0.7796	0.8858	0.8689
TCN_strided_2 × 2	RAW	5.5439	9.1805	0.7224	0.8520	0.8438	5.4744	8.9786	0.7272	0.8542	0.8447
TCN_strided_2 × 2	EWMA	4.8217	7.9291	0.7929	0.8934	0.8805	4.9370	8.1431	0.7756	0.8840	0.8702

#### Attention-based interpretability analysis

Interpretability is especially important in DoA monitoring, where model predictions should reflect meaningful EEG patterns rather than accidental correlations. To examine which parts of the EEG segment contributed most to BIS estimation, we analyzed the attention weights produced by the additive attention pooling layer on the random segment-level test set. Because this layer assigns a normalized importance weight to each time point within a 5-s EEG segment, it enables post hoc analysis of how different temporal regions influence the final prediction.

**Fig. 8:**
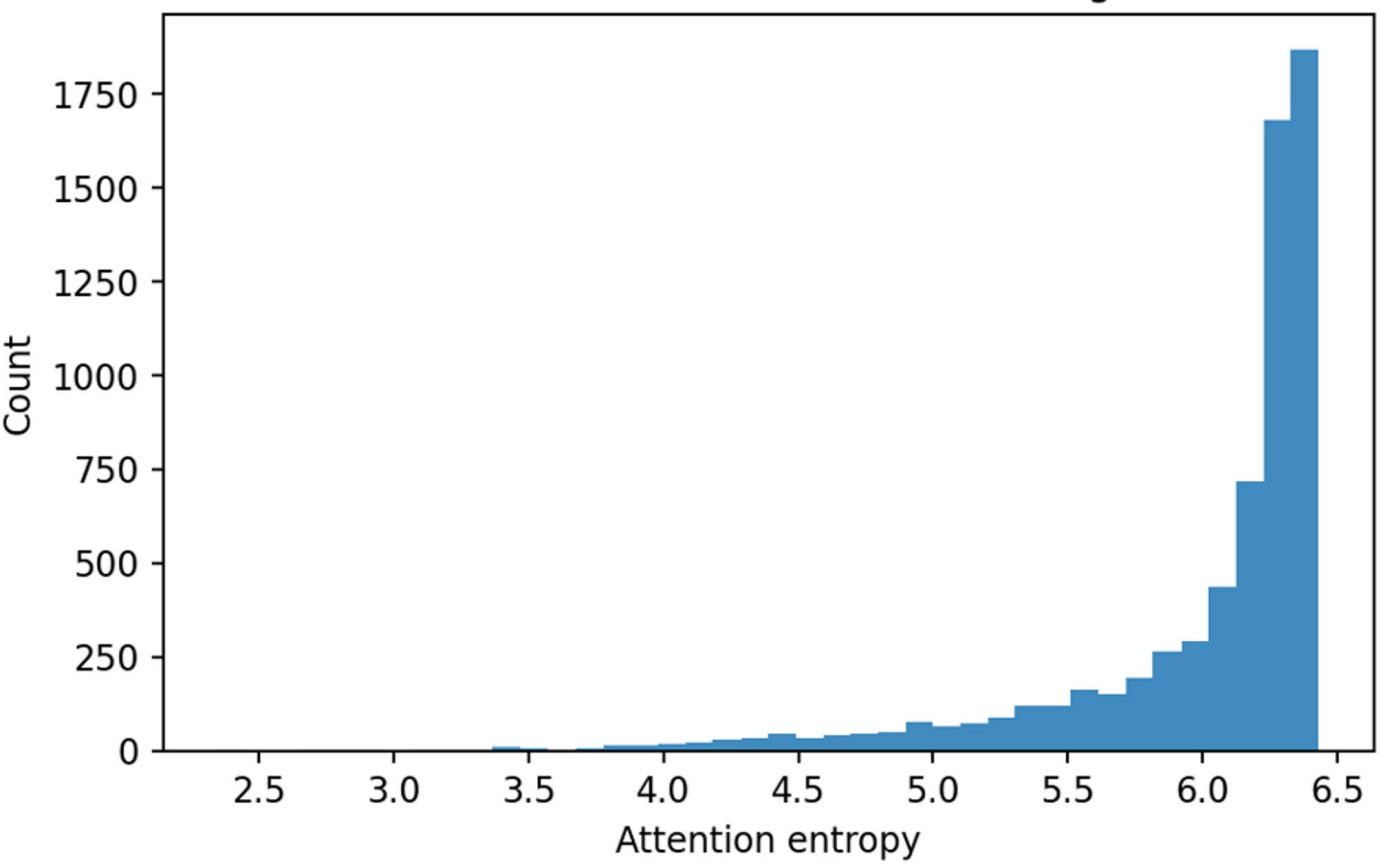
Attention entropy distribution across test segments.

Figure 8 shows the distribution of attention entropy across the test segments. Attention entropy quantifies how broadly or narrowly the attention weights are distributed over time: higher entropy indicates that attention is spread across a larger portion of the segment, whereas lower entropy indicates greater concentration on a smaller number of time points. The distribution is strongly concentrated near the upper end of the observed range, indicating that the model generally relies on broadly distributed temporal information rather than a few isolated samples. This behavior is consistent with BIS being a slowly varying indicator of cortical state rather than a quantity determined by a single brief EEG event.

**Fig. 9:**
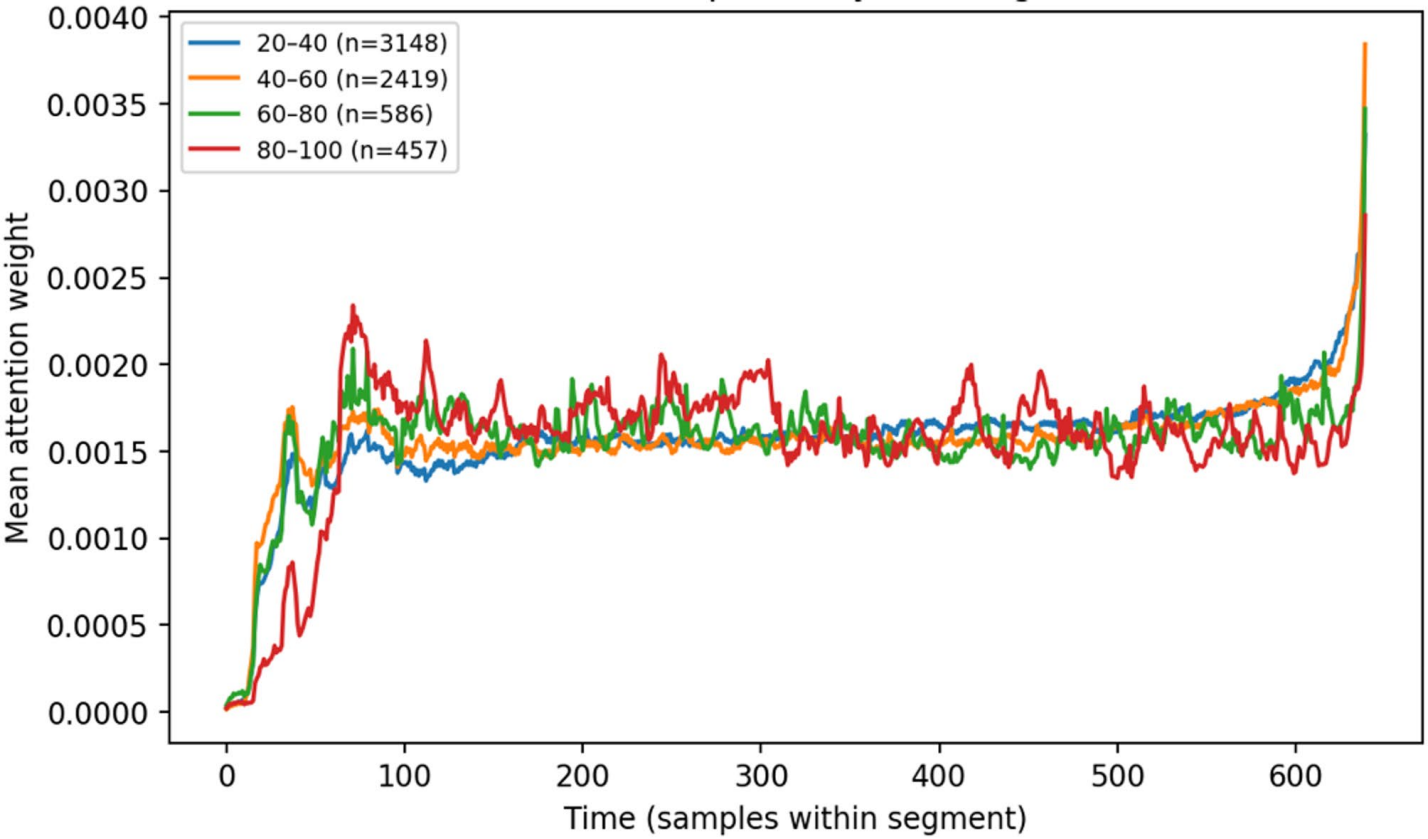
Mean attention profiles over time for different BIS ranges on the test set.

To further examine whether the model changes its temporal focus across anaesthetic states, Fig. 9 shows the mean attention profiles grouped by BIS range. Overall, the profiles are broadly similar across BIS ranges, suggesting that the model uses a largely distributed temporal weighting pattern throughout the segment. At the same time, modest differences are visible: lower BIS ranges appear somewhat smoother, whereas higher BIS ranges show slightly greater temporal fluctuation in the mean attention profile. These differences suggest limited BIS-range-dependent variation in temporal weighting, although the effect appears modest at the level of the averaged curves.

Across all BIS ranges, the model places somewhat greater attention on the later portion of the 5-s EEG segment, with the clearest emphasis occurring near the segment end. One possible interpretation is that more recent EEG activity within the trailing window contributes more strongly to BIS estimation. However, this pattern may also reflect architectural bias. Because the TCN front end is causal and prediction is performed over a fixed trailing window, later inputs may naturally have greater influence on the learned representation. The subsequent BiLSTM–attention stage then operates on features that have already been shaped by this temporal structure. Accordingly, the stronger weighting of later time points should be interpreted cautiously and should not be taken as direct physiological evidence on its own.

Overall, these analyses suggest that the attention mechanism captures structured temporal weighting patterns relevant to BIS estimation while remaining broadly distributed across the segment. At the same time, the interpretability findings should be regarded as supportive rather than conclusive. Additional comparisons with alternative architectures and attribution methods would be needed to determine more clearly whether the observed weighting patterns reflect physiological EEG dynamics, architectural bias, or a combination of both.

.

### Subject-independent evaluation

To more rigorously assess generalization to unseen subjects, we evaluated the proposed BIS estimation framework using a subject-independent protocol based on 5-fold GroupKFold cross-validation, in which all EEG segments from a given subject were assigned exclusively to a single fold. Under this protocol, models were trained and evaluated across five non-overlapping subject folds, and performance metrics were averaged across folds. EWMA smoothing was applied in a subject-wise manner within each fold, with the smoothing parameter α selected independently on the corresponding validation split. This evaluation setting more closely reflects practical deployment scenarios, where BIS estimation models are applied to patients not observed during training and where inter-subject variability in EEG dynamics is expected.

Table 6 reports the mean and standard deviation of performance metrics across folds for three temporal convolution kernel sizes (k = 3, 5, and 7), for both RAW and EWMA-smoothed predictions. Compared to the random segment-level evaluation, subject-independent testing yields higher prediction error across all configurations, reflecting the increased difficulty of generalizing across subjects with heterogeneous EEG characteristics. Nevertheless, performance remains stable across folds, as indicated by relatively modest variability in the reported metrics. Across all kernel sizes, EWMA post-processing consistently improves prediction accuracy and agreement under subject-independent evaluation. Specifically, EWMA reduces MAE and RMSE while increasing both Pearson’s correlation coefficient (r) and CCC relative to RAW predictions in every configuration. For k = 3, MAE decreases from 7.12 ± 0.42 (RAW) to 6.03 ± 0.38 (EWMA), RMSE decreases from 11.26 ± 0.97 to 9.35 ± 1.11, Pearson’s r increases from 0.725 ± 0.042 to 0.816 ± 0.048, and CCC increases from 0.751 ± 0.047 to 0.819 ± 0.050. Similar improvements are observed for k = 5 and k = 7, demonstrating that the stabilizing effect of causal EWMA smoothing generalizes beyond the segment-level setting.

Among the evaluated kernel sizes, k = 3 with EWMA achieves the lowest average MAE and highest CCC under subject-independent cross-validation, while k = 7 yields comparable performance. In contrast, the k = 5 configuration exhibits slightly higher error and greater fold-to-fold variability. This difference suggests increased sensitivity of intermediate receptive-field sizes to inter-subject variation in EEG characteristics when generalizing to unseen patients, despite their favorable performance under random segment-level evaluation. Across folds, the EWMA smoothing parameter α selected on validation data consistently falls within a narrow range, most frequently taking values of 0.05 or 0.10. This concentration indicates a preference for stronger temporal smoothing under unseen-subject evaluation and reflects the increased variability and noise encountered when models are applied to new subjects. Unlike the random segment-level setting, where a single global α enables sensitivity analysis, α selection in the subject-independent protocol is fold-specific, precluding a meaningful global α-sensitivity curve.

Taken together, these results demonstrate that the proposed BIS estimation framework maintains robust performance under a strict subject-independent evaluation protocol. Despite increased prediction error relative to segment-level testing, EWMA smoothing consistently improves accuracy and agreement, and the model exhibits stable behavior across folds. These findings support the applicability of the proposed approach to real-world clinical scenarios involving previously unseen patients.

**Table 6:** Subject-independent performance across 5-fold GroupKFold cross-validation.

Kernel (k)	Post-proc	MAE	RMSE	Pearson’s *r*	CCC
3	RAW	7.12 ± 0.42	11.26 ± 0.97	0.725 ± 0.042	0.751 ± 0.047
3	EWMA	6.03 ± 0.38	9.35 ± 1.11	0.816 ± 0.048	0.819 ± 0.050
5	RAW	7.42 ± 0.85	11.82 ± 1.94	0.712 ± 0.050	0.729 ± 0.083
5	EWMA	6.44 ± 0.81	9.87 ± 1.95	0.803 ± 0.077	0.799 ± 0.080
7	RAW	7.28 ± 0.44	11.27 ± 1.01	0.718 ± 0.046	0.748 ± 0.057
7	EWMA	6.25 ± 0.38	9.36 ± 1.02	0.810 ± 0.067	0.816 ± 0.053

### Statistical comparison of kernel sizes

To assess whether the observed differences among temporal convolution kernel sizes were statistically meaningful, per-subject test-set MAE was compared across models with * k=3*, * k=5*, and * k=7*using a Friedman test followed by pairwise Wilcoxon signed-rank tests with Holm correction. In the random segment-level setting, no statistically significant difference among kernel sizes was observed for RAW predictions $\:{\chi\:}^{2}\left(2\right)=1.33,{\hspace{0.17em}}p=0.513$. Pairwise Wilcoxon signed-rank tests were likewise not significant after Holm correction. The mean per-subject MAE values were 5.83 for * k=3*, 6.00 for * k=5*, and 5.91 for * k=7*, indicating only small patient-level differences before smoothing. For EWMA-smoothed predictions in the random segment-level setting, the omnibus Friedman test was significant $\:{\chi\:}^{2}\left(2\right)=6.33,{\hspace{0.17em}}p=0.042$. Post hoc Wilcoxon signed-rank tests with Holm correction showed that * k=3*significantly outperformed $\:k=7\left(W=54,{\hspace{0.17em}}{p}_{\mathrm{H}\mathrm{o}\mathrm{l}\mathrm{m}}=0.014\right)$, whereas the comparison between * k=3* and * k=5* did not remain significant after correction $\:\left({p}_{\mathrm{H}\mathrm{o}\mathrm{l}\mathrm{m}}=0.121\right)$, and neither did the comparison between * k=5* and $\:k=7\left({p}_{\mathrm{H}\mathrm{o}\mathrm{l}\mathrm{m}}=0.303\right)$. The lowest mean per-subject MAE was observed for * k=3*(4.86), followed by * k=7* (5.07) and * k=5* (5.15). Thus, although the aggregate segment-level metrics in Table 4 favored * k=5*, the paired patient-level statistical analysis in the random segment-level setting showed significant separation only between * k=3*and * k=7* after smoothing.

In the subject-independent 5-fold evaluation, no statistically significant difference among kernel sizes was detected for either RAW or EWMA-smoothed predictions. For RAW predictions, the omnibus Friedman test yielded $\:{\chi\:}^{2}\left(2\right)=1.75,{\hspace{0.17em}}p=0.417$, and none of the pairwise Wilcoxon comparisons remained significant after Holm correction. The corresponding mean per-subject MAE values were 7.65 for * k=3*, 8.27 for * k=5*, and 7.91 for * k=7*. A similar pattern was observed for EWMA-smoothed predictions, for which the omnibus Friedman test yielded $\:{\chi\:}^{2}\left(2\right)=1.58,{\hspace{0.17em}}p=0.453$. Again, none of the pairwise Wilcoxon comparisons remained significant after Holm correction. Nevertheless, * k=3* again showed the lowest mean per-subject MAE (6.31), compared with 7.07 for * k=5* and 6.70 for * k=7*. Taken together, these analyses indicate that the effect of kernel size on patient-level BIS prediction was modest within the evaluated range of $\:k\in\:\{\mathrm{3,5},7\}$, particularly under the stricter unseen-subject protocol. Although * k=3* produced the lowest mean per-subject MAE across both evaluation settings and for both RAW and EWMA-smoothed outputs; this numerical advantage was small and was not consistently supported by statistically significant pairwise differences. Overall, the results suggest limited sensitivity to the precise kernel choice within the tested range, rather than clear and robust superiority of any single kernel size.

### Comparison with prior studies

Table 7 separates prior studies using the same 24-subject dataset from those evaluated on different datasets or cohorts, enabling a clearer and fairer comparison. Although variations in preprocessing, channel configuration, validation protocol, and reported metrics still limit strict head-to-head interpretation, the proposed single-channel end-to-end model remains competitive within the same-dataset group while avoiding hand-crafted feature engineering. The proposed method is also reported under both random segment-level and subject-independent evaluation settings for greater transparency.

**Table 7:** Comparison of prior DoA estimation studies and the proposed method.

Study	Subjects	EEG channels	Methodology/Validation Protocol	Key metrics	Key distinction
**Studies using different datasets**					
Schmierer et al.^22^	9	1	SSEDoA (EWT + SE + SODP + linear SVM)/LOSO-CV	*r* = 0.834; κ = 0.809	Signal-processing + SVM classification pipeline
Alsafy & Diykh^21^	37	1	Sliding windows + HDE + community graph feature selection/Random Sampling	*r* = 0.96	Graph-based entropy feature selection
Shin et al.^24^	35	4	PLE/LOSO-CV	R² = 0.84	PLE-based nonlinear BIS index
Chen et al.^25^	56	1	EEG variability + 8 time-frequency features/LOSO-CV	*r* = 0.70–0.74	Variability-driven handcrafted feature model
Afshar et al.^28^	176	2	Inception CNN + BiLSTM + attention/Random Sampling	RMSE = 5.59 ± 1.04; MAE = 4.3 ± 0.87	Deep attention CNN–BiLSTM model
Afshar & Boostani^29^	176	2	ResNet CNN + BiLSTM/Random Sampling	MAE = 4.6–5.0	Residual CNN–BiLSTM model
Zhang et al.^30^	176	2	FEHANet attention + FFT bands/Subject-independent random hold-out split	MSE = 6.18	Spectral attention hybrid network
Ra et al^23^	24	2	Spectral entropy on 10 frequency bands/LOSO-CV	R² = 0.8458 (β–γ band); R² = 0.7312 (β-band)	Band-wise spectral entropy modeling
**Studies using the same 24-subject dataset**					
Liu et al.^20^	24	1	Sample entropy + multivariate EMD + RF/LOSO-CV	*r* = 0.90 ± 0.05; MAE = 5.22 ± 2.12	Entropy decomposition + RF regression
Shahbakhti et al.^26^	24	1	Parameter-free entropy/power/fractal variation + RF/Random Sampling Parameter-free entropy/power/fractal variation + RF/LOSO CV	*r* = 0.83; MAE = 5.3 *r* = 0.80; MAE = 7.1	Parameter-free handcrafted BIS features
Shahbakhti et al.^27^	24	1	Fuzzy entropy fusion (Gaussian + exponential)/Random Sampling	*r* = 0.85; MAE = 5.4	Fuzzy-entropy feature fusion
**Proposed model**	24	1	TCN–BiLSTM + attention + EWMA/Random Sampling	MAE = 4.49; *r* = 0.903; CCC = 0.897	End-to-end single-channel TCN–BiLSTM with attention and EWMA
**Proposed model**	24	1	TCN–BiLSTM + attention + EWMA/Subject-independent 5-fold GroupKFold,	MAE = 6.03 ± 0.38; *r* = 0.816 ± 0.048; CCC = 0.819 ± 0.05	Same framework with unseen-subject validation

## Limitations and future work

Despite the encouraging results, several limitations should be acknowledged. First, all experiments were conducted on a single EEG dataset. Although this database has been used in prior DoA studies, reliance on a single dataset limits external validity and does not fully establish cross-dataset robustness. Validation on larger, multi-centre datasets with greater diversity in recording hardware, clinical protocols, and patient populations is therefore necessary.

Second, subject-independent evaluation remains a significant challenge. The increased error under GroupKFold testing indicates that the model generalizes less effectively to unseen subjects than to randomly held-out segments. This is likely due to inter-subject differences in baseline EEG characteristics, artefact patterns, physiological variability, and individual responses to anaesthetic drugs. Future work should therefore examine performance stratified by drug type and demographic factors, as these variables may influence the EEG–BIS relationship and partly explain the reduced generalization to unseen subjects. It will also be important to investigate practical methods for reducing this gap, including lightweight subject-specific calibration based on a short patient-specific recording period, test-time personalization through adaptive normalization or limited parameter updating, and domain-adaptation strategies that reduce sensitivity to subject-specific distribution shifts. These approaches may improve robustness while maintaining the simplicity of the proposed framework.

Third, this study focused on a single frontal EEG lead to maintain compatibility with minimal-setup and consumer-grade monitoring devices. While this improves practical applicability, performance may differ when other frontal leads or multi-channel recordings are available. Extending the framework to flexible multi-channel inputs is therefore a natural next step.

Fourth, the interpretability analysis was limited to the random segment-level setting and should be interpreted cautiously. Although the attention results suggest structured temporal weighting, the greater emphasis on later portions of the EEG segment may reflect not only useful signal information but also architectural bias arising from the causal TCN front-end and fixed-window prediction setup. In addition, we did not investigate whether similar temporal weighting patterns are preserved under subject-independent evaluation. Future work should therefore assess interpretability across unseen subjects and compare alternative temporal encoders to better distinguish physiologically meaningful temporal structure from model-induced effects.

Finally, BIS was used as the sole reference for DoA. Although BIS is widely used in clinical practice, it has known limitations, including sensitivity to certain anaesthetic agents and reduced reliability under some physiological conditions. As a result, model behaviour may differ when aligned with other DoA indices or multimodal clinical references. Future work should therefore include comparative evaluation against multiple DoA measures and, where possible, clinician-annotated endpoints.

## Conclusions

This work demonstrates the feasibility of near-real-time, single-channel BIS estimation using a window-level causal end-to-end learning framework with temporal stabilization. The proposed TCN–BiLSTM–attention architecture captures relevant temporal EEG structure while remaining lightweight and computationally efficient, comprising approximately 1.07 million parameters and supporting high-throughput inference on commodity hardware. EWMA post-processing consistently improved prediction stability and agreement across both the random segment-level and subject-independent evaluation protocols, highlighting its practical value for low-latency monitoring. Although absolute performance decreased under subject-independent evaluation, as expected when generalizing to unseen patients, the framework remained stable across folds, supporting its potential relevance to real-world monitoring scenarios. Architectural and ablation analyses further suggest that robust BIS estimation arises from the complementary contribution of temporal receptive-field design, recurrent refinement, attention-based aggregation, and temporal smoothing. More broadly, the contrast between random segment-level and subject-independent results emphasizes the importance of evaluating EEG-based DoA models under multiple testing protocols, particularly when judging generalization to unseen patients. Taken together, these findings support the promise of low-cost, single-sensor BIS estimation and provide a useful foundation for future work toward scalable, transparent, and clinically deployable DoA monitoring systems.

## Data Availability

The dataset analysed during the current study is publicly available on figshare: Ma, Li (2017). *EEG and BIS raw data*. figshare. Dataset. [https://doi.org/10.6084/m9.figshare.5589841.v1](cited in this paper).

## References

[1] Brown, E. N., Purdon,L. & Van Dort, C. J. General anesthesia and altered states of arousal: A systems neuroscience analysis. *Annu. Rev. Neurosci.* **34**, 601–628. 10.1146/ANNUREV-NEURO-060909-153200 (2011). 21513454 10.1146/annurev-neuro-060909-153200 PMC3390788

[2] Fritz, B. A. et al. Intraoperative electroencephalogram suppression predicts postoperative delirium. *Anesth. Analg.* **122**(1), 234–242. 10.1213/ANE.0000000000000989 (2016). 26418126 10.1213/ANE.0000000000000989 PMC4684753

[3] Purdon,L. et al. Mar., Electroencephalogram signatures of loss and recovery of consciousness from propofol, *Proc. Natl. Acad. Sci. U. S. A.*,110,12, (2013). 10.1073/PNAS.1221180110 10.1073/pnas.1221180110 PMC3607036 23487781

[4] Bowdle, T. A. Depth of anesthesia monitoring. *Anesthesiol. Clin. North Am.* **24**(4), 793–822. 10.1016/j.atc.2006.08.006 (2006). 10.1016/j.atc.2006.08.006 17342965

[5] Biro, J. et al. ‘One size’ doesn’t ‘fit all’: Understanding variability in anesthesia work practices. *Hum. Factors Healthc.* **2**, 100026. 10.1016/J.HFH.2022.100026 (2022).

[6] Zhang, X. S., Roy, R. J. & Jensen, E. W. EEG complexity as a measure of depth of anesthesia for patients. *IEEE Trans. Biomed. Eng.* **48**(12), 1424–1433. 10.1109/10.966601 (2001). 11759923 10.1109/10.966601

[7] Rampil, I. J. A primer for EEG signal processing in anesthesia. *Anesthesiology* **89**(4), 980–1002. 10.1097/00000542-199810000-00023 (1998). 9778016 10.1097/00000542-199810000-00023

[8] Akeju, O. et al. Electroencephalogram signatures of ketamine anesthesia-induced unconsciousness. *Clin. Neurophysiol.* **127**(6), 2414–2422. 10.1016/J.CLINPH.2016.03.005 (2016). 27178861 10.1016/j.clinph.2016.03.005 PMC4871620

[9] Punjasawadwong, Y., Phongchiewboon, A. & Bunchungmongkol, N. Bispectral index for improving anaesthetic delivery and postoperative recovery, *Cochrane Database Syst. Rev.*,no. 6,2014, (2014). 10.1002/14651858.CD003843.PUB3 10.1002/14651858.CD003843.pub3 PMC6483694 24937564

[10] Anderson, R. E. & Jakobsson, J. G. Cerebral state monitor, a new small handheld EEG monitor for determining depth of anaesthesia: A clinical comparison with the bispectral index during day-surgery. *Eur. J. Anaesthesiol.* **23**(3), 208–212. 10.1017/S0265021505002206 (2006). 16430792 10.1017/S0265021505002206

[11] Poon, Y. Y. et al. ‘A real-world evidence’ in reduction of volatile anesthetics by BIS-guided anesthesia. *Sci. Rep.* **10**(1), 1–8. 10.1038/S41598-020-68193-X (2020). 32647181 10.1038/s41598-020-68193-x PMC7347920

[12] Connor, C. W. Open reimplementation of the BIS algorithms for depth of anesthesia. *Anesth. Analg.* **135**(4), 10.1213/ANE.0000000000006119. 10.1213/ANE.0000000000006119 (2022). 10.1213/ANE.0000000000006119 PMC9481655 35767469

[13] Schuller,J., Newell, S., Strickland,A. & Barry, J. J. Response of bispectral index to neuromuscular block in awake volunteers. *Br. J. Anaesth.* **115**, i95–i103. 10.1093/BJA/AEV072 (2015). 26174308 10.1093/bja/aev072

[14] Voss, L. & Sleigh, J. Monitoring consciousness: The current status of EEG-based depth of anaesthesia monitors. *Best Pract. Res. Clin. Anaesthesiol.* **21**(3), 313–325. 10.1016/j.bpa.2007.04.003 (2007). 17900011 10.1016/j.bpa.2007.04.003

[15] Lee, H. C. et al. Data driven investigation of bispectral index algorithm. *Sci. Rep.* **9**(1), 1–8. 10.1038/S41598-019-50391-X (2019). 31551487 10.1038/s41598-019-50391-x PMC6760206

[16] Nsugbe, E. & Connelly, S. Multiscale depth of anaesthesia prediction for surgery using frontal cortex electroencephalography. *Healthc. Technol. Lett.* **9**(3), 43–53. 10.1049/HTL2.12025 (2022). 35662750 10.1049/htl2.12025 PMC9160818

[17] Liu, Q. et al. Spectrum analysis of EEG signals using CNN to model patient’s consciousness level based on anesthesiologists’ experience. *IEEE Access* **7**, 53731–53742. 10.1109/ACCESS.2019.2912273 (2019).

[18] Madanu, R., Rahman, F., Abbod, M. F., Fan, S. Z. & Shieh, J. S. Depth of anesthesia prediction via EEG signals using convolutional neural network and ensemble empirical mode decomposition. *Math. Biosci. Eng.* **18**(5), 5047–5068. 10.3934/MBE.2021257 (2021). 34517477 10.3934/mbe.2021257

[19] Huang, X. et al. Research on artificial intelligence-based computer-assisted anesthesia intelligent monitoring and diagnostic methods in health care. *Neural Comput. Appl.* **37**(12), 7813–7822. 10.1007/S00521-023-08998-9 (2025).

[20] Liu, Q., Ma, L., Fan, S. Z., Abbod, M. F. & Shieh, J. S. Sample entropy analysis for the estimating depth of anaesthesia through human EEG signal at different levels of unconsciousness during surgeries. *PeerJ* **2018**(5), e4817. 10.7717/PEERJ.4817 (2018). 10.7717/peerj.4817 PMC5970554 29844970

[21] Alsafy, I. & Diykh, M. Developing a robust model to predict depth of anesthesia from single channel EEG signal. *Phys. Eng. Sci. Med.* **45**(3), 793–808. 10.1007/S13246-022-01145-Z (2022). 35790625 10.1007/s13246-022-01145-z PMC9448694

[22] Schmierer, T., Li, T. & Li, Y. A novel empirical wavelet SODP and spectral entropy based index for assessing the depth of anaesthesia. *Health Inf. Sci. Syst.* **10**(1), 1–14. 10.1007/S13755-022-00178-8/TABLES/3 (2022). 35685297 10.1007/s13755-022-00178-8 PMC9170862

[23] Ra, J. S., Li, T. & Li, Y. A novel spectral entropy-based index for assessing the depth of anaesthesia. *Brain Inform.* **8**(1), 1–12. 10.1186/S40708-021-00130-8/TABLES/3 (2021). 33978842 10.1186/s40708-021-00130-8 PMC8116386

[24] Shin, H. W. et al. Monitoring of anesthetic depth and EEG band power using phase lag entropy during propofol anesthesia. *BMC Anesthesiol*. **20** (1), 1–10. 10.1186/S12871-020-00964-5/TABLES/2 (Feb. 2020). 10.1186/s12871-020-00964-5 PMC7045415 32102676

[25] Chen, Y. F., Fan, S. Z., Abbod, M. F., Shieh, J. S. & Zhang, M. Electroencephalogram variability analysis for monitoring depth of anesthesia. *J. Neural Eng.* **18**(6), 066015. 10.1088/1741-2552/AC3316 (2021). 10.1088/1741-2552/ac3316 34695812

[26] Shahbakhti, M. et al. Wearable EEG-based depth of anesthesia monitoring: A nonparametric feature set. *IEEE Sens. J.* **24**(11), 18098–18107. 10.1109/JSEN.2024.3390604 (2024).

[27] Shahbakhti, M. et al. Fusing fuzzy entropy with Gaussian and Exponential membership functions outperforms traditional entropy metrics in monitoring the depth of anesthesia using a single frontal EEG channel. *IEEE Sens. Lett.* **8**(3), 1–4. 10.1109/LSENS.2024.3369318 (2024).

[28] Afshar, S., Boostani, R. & Sanei, S. A combinatorial deep learning structure for precise depth of anesthesia estimation from EEG signals. *IEEE J. Biomed. Health Inform.* **25**(9), 3408–3415. 10.1109/JBHI.2021.3068481 (2021). 33760743 10.1109/JBHI.2021.3068481

[29] Afshar, S., Boostani, R. & Two-Stage, A. Deep Learning Scheme to Estimate Depth of Anesthesia from EEG Signals, *27th National and 5th International Iranian Conference of Biomedical Engineering, ICBME 2020*,7–12,(2020). 10.1109/ICBME51989.2020.9319416

[30] Zhang, H., Wu, H., Chen, Q. & Xia, Y. FEHANet: A frequency enhanced hybrid attention network for Bispectral Index Score estimation. *Biomed. Signal Process. Control.* **95**, 106431. 10.1016/J.BSPC.2024.106431 (2024).

[31] Hwang, E. et al. Development of a Bispectral Index score prediction model based on an interpretable deep learning algorithm. *Artif. Intell. Med.* **143**, 102569. 10.1016/J.ARTMED.2023.102569 (2023). 37673590 10.1016/j.artmed.2023.102569

[32] Aziz, M. Z. et al. BCINetV1: Integrating temporal and spectral focus through a novel convolutional attention architecture for MI EEG decoding. *Sensors* **25**(15), 4657. 10.3390/s25154657 (2025). 40807821 10.3390/s25154657 PMC12349355

[33] Jiang, W. B., Zhao, L. M. & Lu, B. L. Large Brain Model for Learning Generic Representations with Tremendous EEG Data in BCI, *12th International Conference on Learning Representations, ICLR*, May 2024, Accessed:23, 2026. [Online]. Available:, May 2024, Accessed:23, 2026. [Online]. Available: (2024). http://arxiv.org/abs/2405.18765

[34] Wang, G. et al. EEGPT: Pretrained transformer for universal and reliable representation of EEG signals. *Adv. Neural Inf. Process. Syst.* **37**, 39249–39280. 10.52202/079017-1239 (2024).

[35] Shi, S. & Liu, W. B2-ViT Net: Broad Vision Transformer Network with Broad Attention for Seizure Prediction. *IEEE Trans. Neural Syst. Rehabil. Eng.* **32**, 178–188. 10.1109/TNSRE.2023.3346955 (2024). 38145523 10.1109/TNSRE.2023.3346955

[36] Chen, L. et al. Brain power mapping with optimized deep learning for EEG-based pilot fatigue detection. *Biomed. Signal Process. Control.* **114**, 109284. 10.1016/j.bspc.2025.109284 (2026).

[37] Li, Z. et al. An LSTM network with neural plasticity for driver fatigue recognition on real roads. *IEEE Trans. Ind. Electron.* **72**(12), 14668–14676. 10.1109/TIE.2025.3585046 (2025).

[38] Sangeetha, S. K. B., Mathivanan, S. K., Mallik, S. & Li, A. Recognition predictive modeling using electroencephalogram. *Brain Heart* **2**(2), 2819. 10.36922/bh.2819 (2024).

[39] Item - EEG. and BIS raw data - figshare - Figshare. Accessed:01, 2026. [Online]. Available: https://figshare.com/articles/dataset/EEG_and_BIS_raw_data/5589841/1

[40] Shalbaf, R., Behnam, H., Sleigh, J. W., Steyn-Ross, A. & Voss, L. J. Monitoring the depth of anesthesia using entropy features and an artificial neural network. *J. Neurosci. Methods.* **218**(1), 17–24. 10.1016/j.jneumeth.2013.03.008 (2013). 23567809 10.1016/j.jneumeth.2013.03.008

[41] Bai, S., Kolter, J. Z. & Koltun, V. An Empirical Evaluation of Generic Convolutional and Recurrent Networks for Sequence Modeling,Accessed:04, 2025. [Online]. (2018). Available: https://arxiv.org/pdf/1803.01271

[42] Wang, Y. et al. Short-term load forecasting for industrial customers based on TCN-LightGBM. *IEEE Trans. Power Syst.* **36**(3), 1984–1997. 10.1109/TPWRS.2020.3028133 (2021).

[43] Fan, J., Zhang, K., Huang, Y., Zhu, Y. & Chen, B. Parallel spatio-temporal attention-based TCN for multivariate time series prediction. *Neural Comput. Appl.* **35**(18), 13109–13118. 10.1007/S00521-021-05958-Z/METRICS (2023).

[44] Zhao, Y., Ren, J., Zhang, B., Wu, J. & Lyu, Y. An explainable attention-based TCN heartbeats classification model for arrhythmia detection. *Biomed. Signal Process. Control* **80**, 104337. 10.1016/J.BSPC.2022.104337 (2023).

[45] Al-qaness, M. A. A., Dahou, A., Trouba, N. T., Elaziz, M. A. & Helmi, A. M. TCN-Inception: Temporal convolutional network and Inception modules for sensor-based human activity recognition. *Future Gener. Comput. Syst.* **160**, 375–388. 10.1016/J.FUTURE.2024.06.016 (2024).

